# Characterization of Variable Region Genes and Discovery of Key Recognition Sites in the Complementarity Determining Regions of the Anti-Thiacloprid Monoclonal Antibody

**DOI:** 10.3390/ijms21186857

**Published:** 2020-09-18

**Authors:** Pengyan Liu, Yuanhao Guo, Shasha Jiao, Yunyun Chang, Ying Liu, Rubing Zou, Yihua Liu, Mengli Chen, Yirong Guo, Guonian Zhu

**Affiliations:** 1 Institute of Pesticide and Environmental Toxicology, Ministry of Agriculture Key Laboratory of Molecular Biology of Crop Pathogens and Insects, Zhejiang University, Hangzhou 310058, China; lpyainimen@163.com (P.L.); 21916108@zju.edu.cn (Y.G.); 21616183@zju.edu.cn (S.J.); 21716193@zju.edu.cn (Y.C.); 21416116@zju.edu.cn (Y.L.); zourubing@zju.edu.cn (R.Z.); cmlmeng@126.com (M.C.); zhugn@zju.edu.cn (G.Z.); 2 Department of Food Science and Nutrition, Zhejiang Key Laboratory for Agro-Food Processing, Zhejiang University, Hangzhou 310058, China; 3 Research Institute of Subtropical Forestry, Chinese Academy of Forestry, Hangzhou 311400, China; 4 Zhejiang Provincial Key Laboratory of Biometrology and Inspection & Quarantine, College of life sciences, China Jiliang University, Hangzhou 310018, China

**Keywords:** LC-MS/MS, next-generation sequencing, recombinant full-length IgG, sequences of variable region genes, recognition sites, immunoassay, thiacloprid

## Abstract

Sequence-defined recombinant antibodies (rAbs) have emerged as alternatives to hybridoma-secreted monoclonal antibodies (mAbs) for performing immunoassays. However, the polyploidy nature of hybridomas often leads to the coexistence of aberrant or non-specific functional variable region (VR) gene transcripts, which complicates the identification of correct VR sequences. Herein, we introduced the use of LC-MS/MS combined with next-generation sequencing to characterize VR sequences in an anti-thiacloprid mAb, which was produced by a hybridoma with genetic antibody diversity. The certainty of VR sequences was verified by the functional analysis based on the recombinant antibody (rAb) expressed by HEK293 mammalian cells. The performance of the rAb was similar to that of the parental mAb, with IC_50_ values of 0.73 and 0.46 μg/L as measured by ELISAs. Moreover, molecular docking analysis revealed that Ser52 (H-CDR2), Trp98, and Trp93 (L-CDR3) residues in the complementarity determining regions (CDRs) of the identified VR sequences predominantly contributed to thiacloprid-specific recognition through hydrogen bonds and the CH–π interaction. Through single-site-directed alanine mutagenesis, we found that Trp98 and Trp93 (L-CDR3) showed high affinity to thiacloprid, while Ser52 (H-CDR2) had an auxiliary effect on the specific binding. This study presents an efficient and reliable way to determine the key recognition sites of hapten-specific mAbs, facilitating the improvement of antibody properties.

## 1. Introduction

As alternatives to the classical instrumental methods, immunoassays are recognized as rapid, simple, and cost-effective analytical techniques for on-site screening of small molecular contaminants in environmental and agricultural samples. It is well known that an accurate and reliable immunoassay depends on the core reagent, namely an antibody with high sensitivity, specificity, and stable performance.

Neonicotinoid insecticides are among the most extensively used agrichemicals in the world. However, in recent years, they have gradually attracted people’s concerns regarding their adverse effects on non-target organisms, such as bees [1], spiders [2], and mammals [3,4]. Since thiacloprid is one of the representative varieties of these insecticides, which has been banned for outdoor use in the EU since January 2020 [5], it is urgent to establish sensitive and quick analytical methods for reliable monitoring of thiacloprid residues in environmental and agricultural samples. Thus, the investigation of antibodies against thiacloprid is the priority for the immunoassay development.

In our previous study, a hybridoma secreting monoclonal antibody (mAb) against thiacloprid was produced and used to develop a sensitive electrochemical immunosensing method, with a detection limit (LOD) of 0.1 μg/L [6]. Unfortunately, we found that the performances of ascite monoclonal antibodies (mAbs) were different from batch to batch, which directly affected the reproducibility of the assay sensitivity. Likewise, this issue had been reported by many other researchers. They announced that some antibodies failed to meet the standards as expected, such as unspecific binding toward non-target analytes [7,8] and insufficient inter-batch stability and reproductivity [9]. Additionally, the unstable performance of hybridoma-derived mAbs hinders their large-scale application due to the deterioration or loss of hybridoma cell lines. To solve these bottlenecks of the traditional mAbs, Andrew Bradbury and 110 cosignatories proposed that antibodies for diagnosis must be produced as sequence-defined recombinant antibodies (rAbs), given the improved sensitivity and specificity and the dramatically improved reproducibility and stability achieved with appropriate expression systems [10,11]. Therefore, accurate characterization of the functional and specific heavy-chain (VH) and light-chain (VL) variable region (VR) sequences from mAb-producing hybridomas is the key to generating desirable rAbs, which also benefits the further discovery of the key recognition amino acids in complementarity-determining regions (CDRs) responsible for hapten-specific binding [12]. 

Usually, PCR amplification coupled with Sanger sequencing is used for the acquisition of antibody VR genes derived from hybridoma cell lines [13,14]. However, PCR amplification of specific functional VRs transcripts is often complicated and hindered by the coexistence of aberrant immunoglobulin (Ig)-like and non-specific functional VR gene transcripts [15,16], owing to the polyploidy nature of hybridomas [17]. Sometimes, the functional IgG sequences found in hybridomas are identical to the defined VR sequences previously reported in the NCBI for unique antibodies [12]. Therefore, multiple PCR amplifications and in vitro expression of various VH–VL combinations are necessary to identify the correct VHs and VLs in appropriate pairings, but this strategy is obviously inefficient and laborious for a large number of hybridoma samples.

Liquid chromatography coupled with tandem mass spectrometry (LC-MS/MS) is a powerful high-resolution technology that is becoming more important in peptide and protein identification and mAb analysis [18,19]. It is particularly beneficial for meeting the important needs of the biopharmaceutical industry and characterizing modifications with small mass changes, which significantly enhanced our capability to explain the characterization of the mAb [20,21]. De novo protein sequencing based on LC-MS/MS is considered a complementary method to VR gene sequencing [22,23]. Nevertheless, there are still some difficulties and technical bottlenecks, such as difficulty in distinguishing isoleucine or leucine [24] and equivocality in spectral interpretation. Moreover, de novo assembly of unknown mAb sequences could be a challenge. Since each Ig VR domain shows its own unique variability, de novo antibody sequencing is rather tough and merely predictive if used without an authentic gene sequence map [12,25]. 

In recent years, high-throughput immune repertoire sequencing has sprung up rapidly as a valuable technique for obtaining large-scale information on antibody repertoire diversity, investigating the evolution process of functional antibody repertoires [26], direct discovery of new antibodies, and generation of rAbs [27]. Based on next-generation sequencing (NGS) technology, the entire VH and VL clone types of hybridomas and their relative abundances can be clearly revealed [17,28]. Until now, NGS-based antibody repertoire analysis has not been applied in the sequence characterization of hapten-specific antibodies against small molecular pollutants.

Herein, this study highlights the importance of an efficient and accurate approach for the characterization of thiacloprid-specific VR sequences from a hybridoma with genetic antibody diversity through LC-MS/MS assisted with NGS and specific PCR. The certainty of the identified VR sequences was verified by the function of a full-length “Y-shaped” IgG expressed in the HEK 293 (F) mammalian cell system, which mimics the natural mouse-derived mAb, instead of common recombinant antibody (rAb) fragments or inadequate *Escherichia coli* (*E. coli*) expression systems [12,29,30]. Based on the characterized VH and VL gene sequences, the 3D homology modeling and molecular docking were performed to investigate the thiacloprid antibody recognition mechanism. The discovered key amino acids for specific binding were confirmed by single-site-directed alanine mutagenesis.

## 2. Results

### 2.1. Recognition Features of the mAb Measured by Surface Plasmon Resonance (SPR)

#### 2.1.1. The mAb Selectivity 

At the end of the coupling program for the surface plasmon resonance (SPR) immunosensor chip, the mAb immobilization level reached 29,583 resonance units (RU) (Figure S1). In order to identify the recognition spectrum of the mAb-C4C4, low molecular weight (LMW) selectivity screening was performed by the direct SPR immunoassay. The results (Figure S2) showed the binding abilities of anti-thiacloprid mAb toward five neonicotinoid pesticides. It was observed that thiacloprid and imidaclothiz displayed high binding responses, while imidacloprid, acetamiprid, and clothianidin showed medium binding responses. No significant binding was found toward dinotefuran, nitenpyram, and thiamethoxam, similar to the negative controls of two organophosphorus pesticides.

#### 2.1.2. Kinetics and Affinity of the mAb

**Figure 1:**
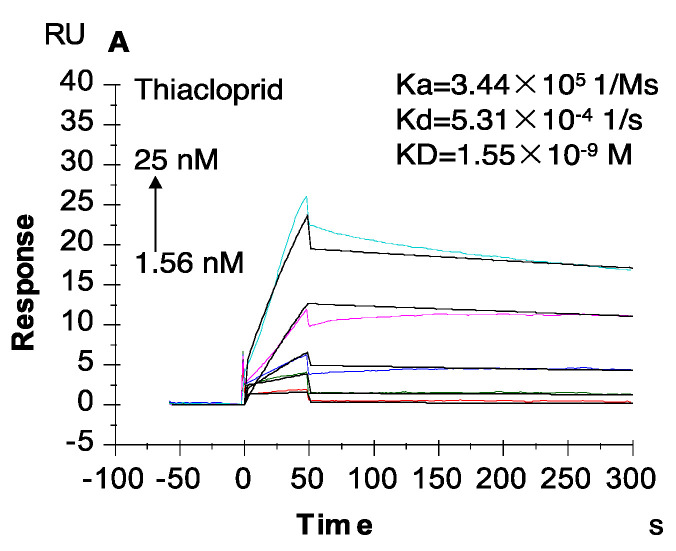
Kinetics and affinity of the monoclonal antibody (mAb) with thiacloprid measured by surface plasmon resonance (SPR). Resonance units (RU) were double-subtracted by the reference flow cell signal and the buffer control. Ka: association rate; Kd: dissociation rate; KD: dissociation equilibrium constant.

To evaluate the dynamic binding kinetics and affinity of the mAb to the compounds, real-time SPR biosensing experiments were conducted. The final double referenced SPR sensorgrams were fitted to a simple 1:1 interaction model, which determined the association rate (Ka), dissociation rate (Kd), and dissociation equilibrium constant (KD = Kd/Ka) values. The results showed that the association–dissociation kinetic modes of the anti-thiacloprid mAb toward various compounds could be divided into three categories (Figure 1 and Figure S3): (1) Thiacloprid and acetamiprid belong to the “fast on and rare off” group (Figure 1A and Figures S3B), meaning that the formed immunocomplexes were hard to dissociate (Kd of 5–6 × 10^−4^ 1/s) after the quick binding reaction. The dissociation equilibrium constant value of KD = 1.55 × 10^−9^ M for thiacloprid was obviously lower than KD = 2.39 × 10^−9^ M for acetamiprid, suggesting that the mAb exhibited a higher affinity to thiacloprid than to acetamiprid. (2) Imidacloprid, imidaclothiz, and clothianidin were considered as the “fast on and slow off” group (Figure S3C–E), i.e., quick binding was followed closely by 600 s of slow dissociation reaction with a Kd value of 1–4 × 10^−2^ 1/s, and almost all of the complexes finally dissociated at the end of 600 s, with KD values close to 10^−8^ M. Therefore, the affinity of imidaclothiz and imidacloprid was still lower than that of thiacloprid and acetamiprid, although they had high binding signals in the LMW selectivity screening assay. (3) Dinotefuran, nitenpyram, and thiamethoxam exhibited the “fast on and off” behavior (Figure S3F–H), revealing that complete dissociation happened after the binding reaction, which immediately reverted to the baseline. The SPR immunosensor results indicated that the anti-thiacloprid mAb displayed high selectivity and sensitivity for thiacloprid, which could be considered as a satisfying source for the development of rAbs.

### 2.2. Sequence Diversity and Abundance of VR genes

#### 2.2.1. VR Gene Clone Types and Abundances 

**Figure 2:**
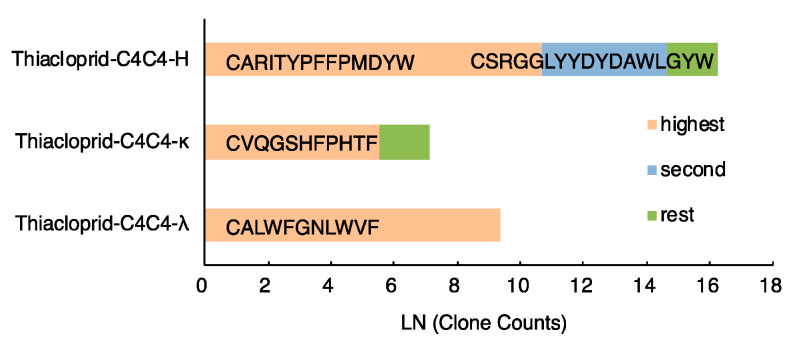
The clone types and abundances of variable regions of heavy chain, λ light chain and κ lignt chain (VH, Vλ, and Vκ) in the anti-thiacloprid hybridoma C4C4 measured by next-generation sequencing (NGS).

The anti-thiacloprid hybridoma C4C4 produced 2037 megabase (Mb) with NGS, with 86.04% of these satisfying Q ≥ 30. The raw sequencing results were adjusted by unique molecular barcodes (UMBs) to correct the errors from PCR amplification and sequencing procedures. As the most diverse region, CDR3 contains V-(D)-J segments and is often used to identify different clones. Figure 2 and Table S1 show that the thiacloprid-C4C4-lambda (λ) library produced 11,712 transcripts, all of which had the same CDR3 amino acid sequence “CALWFGNLWVF”, while the thiacloprid-C4C4-kappa (κ) library only produced 252 transcripts, far less than thiacloprid-C4C4-λ, 247 of which shared the same CDR3 amino acid sequence “CVQGSHFPHTF”. The thiacloprid-C4C4-H library produced 45,243 transcripts, 99.88% of which had the same CDR3 sequence “CARITYPFFPMDYW”, with the clone “CSRGGLYYDYDAWLGYW” having only 0.11% abundance at the second position. 

#### 2.2.2. Accurate Full-Length Sequences of Interested VH and VL Clones

**Figure 3:**
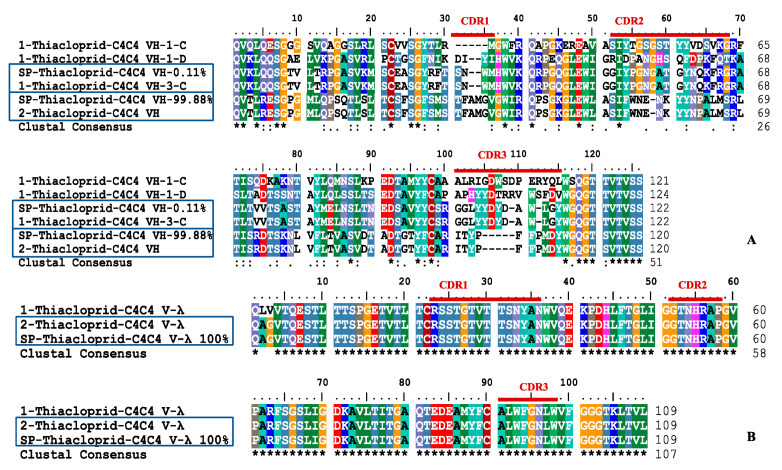
VH (**A**) and V-λ (**B**) amino acid sequences of anti-thiacloprid hybridomas aligned by BioEdit software and three complementarity determining regions (CDRs) classified by abYsis.

The full-length nucleotide sequences of amplified VH and VL products were analyzed by ImMunoGeneTics information system (IMGT). All the sequences were V-(D)-J in frame, translated (functional), and without an early termination codon. Three CDRs of VH and VL were annotated according to the Kabat rule (Figure 3).

Using specific primers designed according to the NGS results, the accurate full-length sequences of our interested clones were amplified and named as “SP-thiacloprid-C4C4 VH-99.88% (specific primer amplified full-length sequence of the VH clone with 99.88% abundance)”, “SP-thiacloprid-C4C4 VH-0.11% (specific primer amplified full-length sequence of the VH clone with 0.11% abundance)”, and “SP-thiacloprid-C4C4 V-λ 100% (specific primer amplified full-length sequence of the V-λ clone with 100% abundance)”, respectively. In the comparative experiment, using primer set 1, we obtained three VH sequences and one VL sequence, named as “1-thiacloprid-C4C4”. Using primer set 2, we obtained each one sequence of VH and VL, named as “2-Thiacloprid-C4C4”. Translated amino acid sequences were aligned separately by Protein BLAST in NCBI database. The sequence homology showed that most of those sequences were highly homologous with Ig VHs or VLs of mice, except that “1-thiacloprid-C4C4 VH-1-C” was highly homologous with the Ig VH of *Camelus bactrianus*.

Amino acid sequences were aligned in each group by BioEdit software. It was found that the 6 VH sequences showed significant variety with a low identity (Figure 3A). SP-Thiacloprid-C4C4 VH-99.88% was the full-length sequence of the related clone “CARITYPFFPMDYW”, which had 99.88% abundance in the thiacloprid-C4C4-H NGS library and was identical to “2-Thiacloprid-C4C4 VH” amplified by primer set 2. SP-Thiacloprid-C4C4 VH-0.11% was the full-length sequence of the related clone “CSRGGLYYDYDAWLGYW”, with only 0.11% abundance in the second position in the thiacloprid-C4C4-H NGS library, which was identical to “1-thiacloprid-C4C4 VH-3-C” amplified by primer set 1. The identification of VL was more than 97% (Figure 3B)—only 2 amino acids at the beginning of frame region 1 (FR1) were different. SP-Thiacloprid-C4C4 V-λ 100% was the full-length sequence of the related clone “CALWFGNLWVF”, with 100% abundance being sequenced by NGS in the thiacloprid-C4C4-λ library, which was exactly the same as “2-Thiacloprid-C4C4 V-λ”. 

### 2.3. Determination of VR Sequences of the mAb

The peptides of the mAb identified by LC-MS/MS (Figure S4) were assembled and mapped to the established “antibody VR sequence database” by PEAKS Studio X. The mAb achieved 100% sequence coverage for the predicted VH sequence “SP-thiacloprid-C4C4 VH-99.88%” (120/120) and VL sequence “SP-thiacloprid-C4C4 V-λ 100%” (109/109), which were obtained from NGS of the antibody transcriptome and specific PCR (Figure S5), which failed to completely overlap the predicted VH sequence “SP-thiacloprid-C4C4 VH-0.11%”. Taken together, we can draw a conclusion that “SP-thiacloprid-C4C4 VH-99.88%” and “SP-thiacloprid-C4C4 V-λ 100%” were the productive specific VR sequences of anti-thiacloprid mAb, while the clone with low coverage and fewer clone counts seemed to be the redundant contaminated sequence.

### 2.4. Performance of the Full-Length rAb and the Parental mAb 

#### 2.4.1. Selectivity and Sensitivity to Neonicotinoid Pesticides

**Figure 4:**
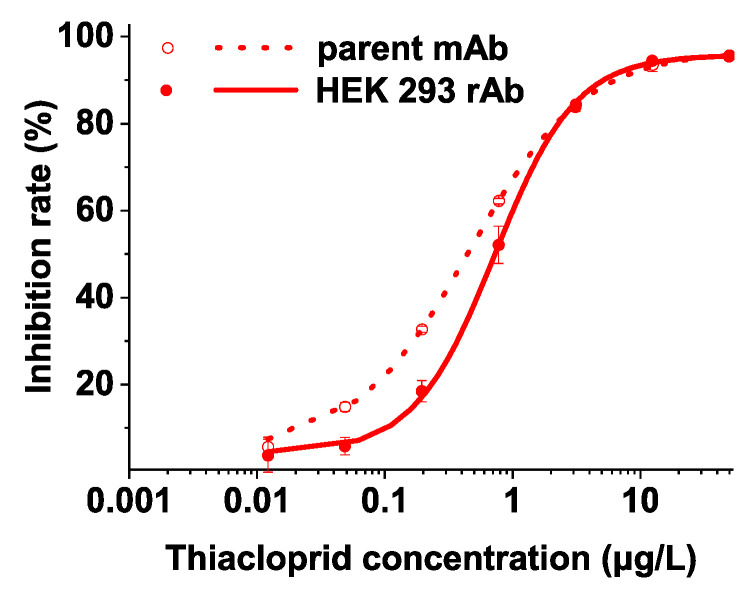
Standard curves of thiacloprid measured by IC-ELISA based on the ascite mAb and the rAb expressed by HEK 293(F) cells.

Functional verification of the predicted VH and VL sequences was performed by using full-length rAb expressed in HEK 293(F) mammalian cells, as shown in the Supplementary Materials for detailed results of antibody expression (Figures S6 and S7). Indirect competitive ELISA (IC-ELISA) was performed to analyze the specificity and sensitivity of the full-length rAb and the parental mAb. The results from triplicate determinations were fitted by the four-parameter equation with the correlation coefficient (R^2^) > 0.99. Thiacloprid-C4C4 mAb had the highest recognition activity toward thiacloprid, with IC_50_ of 0.46 μg/L and a linear range of 0.11 to 1.94 μg/L (Figure 4). This was consistent with the results obtained by SPR method, i.e., the mAb exhibited the highest affinity to thiacloprid. The thiacloprid-C4C4 mAb displayed obvious cross-reactivity (CR) with acetamiprid (35.67%), slight CR with 2-chloro-5-[[2-(nitromethy-l-ene)-1-imidazolidinyl] methyl] pyridine (6-Cl-PMNI) and the thiacloprid amide (around 2%), and no obvious CR (<2%) with other structural analogues (Figure S8). There were some inconsistencies in the recognition order of mAb with the pesticides between IC-ELISA and SPR results, probably because ELISA included the competitive reaction of 1 h of incubation in order to reach the equilibrium, while SPR assay involved the non-competitive immunoreaction in a flow system containing the association and dissociation phases within a few minutes. 

The standard curves for the full-length rAb were also established by IC-ELISA. The linear range for thiacloprid was 0.27–2.01 μg/L and the IC_50_ was 0.73 μg/L (Figure 4). Additionally, the anti-thiacloprid full-length rAb exhibited a certain CR of 33.32% to acetamiprid, very low CR (3–4%) to 6-CL-PMNI and the thiacloprid amide, and no obvious CR (<2%) with other structural analogues (Figure S8). Thus, the rAb revealed the selectivity and sensitivity close to the parental mAb. Importantly, the full-length rAb prepared in our study showed higher sensitivity compared with the previously reported antibodies against thiacloprid (Table 1), an important analyte in recent years. It can be further used as an effective reagent to develop various immunoassays with high sensitivity for rapid and trace detection of thiacloprid residues in the agro-products and environmental samples. 

#### 2.4.2. Gold Immunochromatographic Strip (GICS) Application

**Figure 5:**
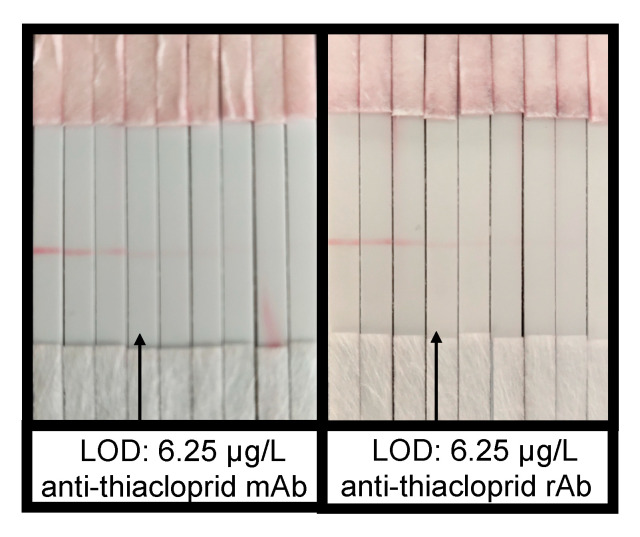
Immunostrip test for a serial solution of thiacloprid at concentrations of 0, 1.56, 3.13, 6.25, 12.50, 25, 50, 100, and 200 μg/L (from left to right).

As a rapid and simple method, GICS was used to verify the performances of the rAb and mAb and to extend applications for one-step detection of pesticide residues. As judged by the naked eye, the LOD values of thiacloprid were both 6.25 μg/L using the strips based on the rAb and the mAb (Figure 5), mirroring again that the rAb had almost the same high affinity as the mAb. The satisfying sensitivity of the rAb-based GICS was ascribed to the fast and stable binding of thiacloprid with the natural mAb, as mentioned above in the SPR kinetic test. Overall, the result demonstrated that the full-length rAb could be a good alternative to the parental mAb and could be used as a popular on-site rapid test in the future. 

### 2.5. The Key Amino Acids in the CDRs Responsible for Specific Binding of Thiacloprid

**Figure 6:**
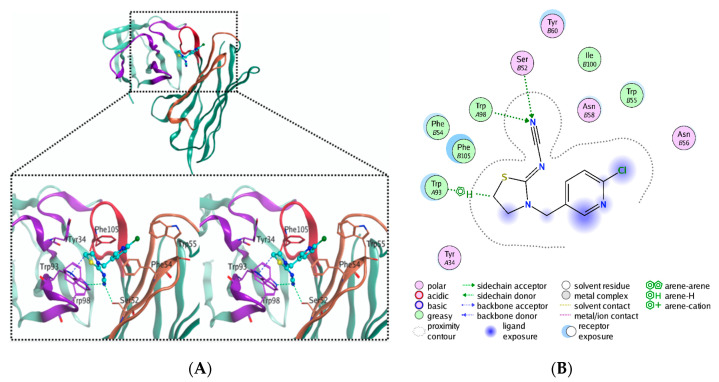
(**A**) The predicted binding mode of thiacloprid to the variable fragment (Fv). The upper part shows a ribbon model of the Fv structure and the 3D structure of the interacted thiacloprid. The lower part shows a portion of the structure of the complex, which is magnified in the black box and displayed with a wall-eye stereo image. Thiacloprid is shown with a ball-and-stick model; the variable fragments (Fvs) are shown in ribbons and colored in dark green (FRs), purple (VL-CDR1/2/3), brown (VH-CDR1/2), and red (VH-CDR3). The residues that interacted with thiacloprid are highlighted as sticks and hydrogen bonds are emphasized by green lines. The CH–π interaction is indicated by a blue line. (**B**) The 2D diagram of the key amino acid residues and main interactions that contributed to the stability of antibody–thiacloprid complex.

High-quality Fv (variable fragment) model was constructed and shown in Supplementary Materials (Figures S9 and S10). The predicted 3D binding mode of thiacloprid to Fv is depicted in Figure 6A. The 2D diagram of the key amino acid residues and main interactions that contributed to the stability of the immunocomplex is displayed in Figure 6B. The molecular docking analysis revealed that thiacloprid was strongly bound by the antibody, with a binding free energy score of –5.857 kcal/mol, mainly through hydrogen bonds, CH-π, and VDW (van der Waals) interactions. Ser52 (H-CDR2), Trp98 (L-CDR3), and Trp93 (L-CDR3) residues, which predominantly contributed to thiacloprid-specific recognition, were successfully discovered. The OH of the Ser52 (H-CDR2, 3.29 Å) residue and indolyl of the Trp98 (L-CDR3, 3.02 Å) residue formed two hydrogen bonds with CN on thiacloprid, respectively; the π electron conjugation system of Trp93 (L-CDR3, 3.65 Å) residue formed an additional CH–π interaction with the tetrahydrothiazole ring of thiacloprid.

**Figure 7:**
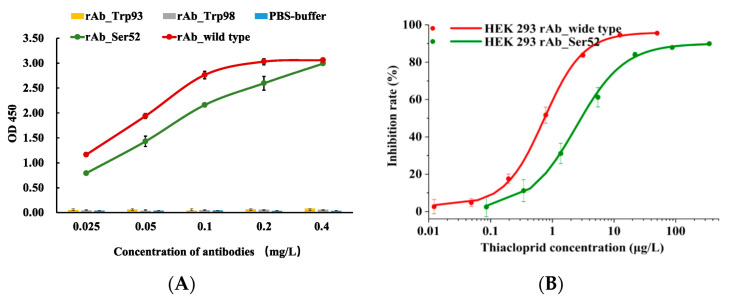
(**A**) Binding analysis of alanine mutants to thiacloprid-OVA evaluated by ELISA. Experiments were performed in triplicate. (**B**) Standard curves of thiacloprid measured by IC-ELISAs based on the rAb_wild-type and the rAb_Ser52 mutant.

The key amino acids were confirmed by means of single-site-directed alanine mutagenesis. The binding activities of 3 single-point mutated rAbs were evaluated by ELISA to confirm the functional effect of the key amino acid residues on antigen–antibody interactions (Figure 7A). The results indicated that 3 alanine mutants could be classified into 2 groups. Compared with the positive control and negative control, replacements of Trp93 (L-CDR3) or Trp98 (L-CDR3) with alanine led to complete loss of the binding ability. In contrast, the Ser52 (H-CDR2)-mutated rAb exhibited binding behavior to the antigen thiacloprid-ovalbumin (OVA) close to that of the wild-type rAb. The degree of influence of the Ser52 (H-CDR2) residue was further investigated by IC-ELISA. The results shown in Figure 7B clearly suggest that the Ser52 (H-CDR2) mutant presented lower recognition sensitivity to thiacloprid, with IC_50_ of 2.43 μg/L, displaying a decrease of nearly 3.33-fold compared to the wild-type rAb. Above all, Trp98 (L-CDR3) and Trp93 (L-CDR3) were verified as necessary amino acids in the conformation of the VL CDRs and were attributed to the high affinity for thiacloprid; Ser52 (H-CDR2) played an auxiliary role in the specific interaction with thiacloprid. Singly altering these residues with alanine residue prevented or decreased the binding affinity of the mutated rAb.

In addition, VDW interactions with the surrounding polar and hydrophobic residues (Phe105, H-CDR3; Phe54, H-CDR2; Ile100, H-CDR3; Trp55, H-CDR2; Asn56, H-CDR2; Tyr34, L-CDR1) also contributed to the recognition.

## 3. Discussion 

A recent study analyzed 185 hybridomas and revealed that 31.9% of them contained one or more additional productive heavy or light chains, which exhibited the genetic diversity of VH and VL genes in individual hybridomas [17]. The contaminative functional sequences (non-specific for antigen recognition) may be derived from a hybridoma that is not absolutely monoclonal, which could be induced by more than one spleen cell fused with myeloma partners [36] or a spleen cell that has been fused but containing a rearranged allele in the excluded chromosomes, a so-called “allelic inclusion” [37,38], along with mutations during the long-term cultivation. The aberrant unproductive VH and VLκ chains may be derived from the myeloma fusion partners caused by Ig VH and VLκ allelic aberrant rearrangement [38,39,40]. 

For a long time, single PCR amplification has been reported as the main method for characterization of the hapten-specific VH and VL sequences from hybridomas with diverse antibody genes. However, the incorrect sequences, such as the non-specific functional or aberrant Ig VR products, were frequently obtained due to the probable off-target amplification [41]. This similar phenomenon was also found in our comparative experiments based on single PCR amplification and Sanger sequencing. Amplified by primer set 1, three functional VH sequences and one functional VL sequence of Thiacloprid-C4C4 hybridoma were obtained. Among the PCR amplified VHs and VL, “1-thiacloprid-C4C4 VH-1-C” was highly homologous with the Ig VH of *Camelus bactrianus*; “1-thiacloprid-C4C4 VH-3-C” was identical to “SP-thiacloprid-C4C4 VH-0.11%”, with only 0.11% abundance at the second position in the thiacloprid-C4C4-H NGS library, while the LC-MS/MS coverage analysis showed the sequence could not be 100% covered, indicating that it was a contaminative sequence that did not necessarily encode the antibody chain with the expected specificity; “1-thiacloprid-C4C4 VH-1-D” was not discovered in the thiacloprid-C4C4-H NGS library, or if the abundance was less than 2.21 × 10^−5^ it could be ignored, while the LC-MS/MS coverage analysis showed it could not be 100% covered and was deemed as another contaminative VH sequence. “1-Thiacloprid-C4C4 V-λ” had two amino acids at the beginning position of FR1 that differed from “SP-thiacloprid-C4C4 V-λ 100%” in the thiacloprid-C4C4-λ NGS library, while the LC-MS/MS coverage analysis showed these two amino acids could not be covered, which were also determined as non-target sequences. Thus, it is necessary to adopt the additional PCR amplifications with alternate primer sets to obtain the target sequences. This process is complicated, time-consuming, and unreliable due to the unclear genetic diversity in hybridomas.

Primer set 2 helped us to amplify “2-Thiacloprid-C4C4 VH” and “2-Thiacloprid-C4C4 V-λ” successfully with limited off-target amplification of confused sequences, confirming these sequences as completely the same as the “SP-thiacloprid-C4C4 VH-99.88%” and “SP-thiacloprid-C4C4 V-λ 100%” sequences obtained by NGS of the antibody transcriptome, and which were further identified as hapten-specific sequences by MS/MS peptide coverage analysis. However, none of one primer sets are suitable for amplifying the correct VRs of all hybridomas. For instance, another study in our laboratory indicated that primer set 2 can also amplify the contaminated VR gene sequences from imidacloprid-C4 hybridoma by PCR amplification, which included 2 functional VH sequences, 1 functional Vλ sequence, and 1 functional Vκ sequence. Therefore, it still seems impossible to figure out whether these sequences contained the correct sequences of VH and VL that could recognize antigens. 

Herein, we suggest the NGS technique based on the antibody transcriptome as an efficient and universal method to deal with various intricate cases, since it can clarify all of the VR gene sequence diversities and gene abundances of individual hybridomas, complemented with specific PCR to amplify exact full-length sequences of the VH and VL. Normally, the clones with the highest amplification frequency are considered to be antigen-specific, i.e., the highly amplified VH and VL genes were paired to construct the rAbs, however subsequent experiments confirmed that the majority of clones were actually antigen-specific [42,43]. However, Bradbury et al. hold the opposite viewpoint that the most abundant transcripts of VH and VL found in a hybridoma do not exactly translate to the antibody displaying the desired sensitivity and specificity [17]. Therefore, it is necessary to express multiple VH–VL combinations to characterize the correct combination of the VH and VL sequences that can specifically recognize the target ligand. Obviously, this strategy is only suitable for the sequence characterization in a small amount of hybridomas, whereas it is inefficient and labor-intensive for a large number of hybridomas. Hence, we propose that when the hybridomas have multiple amino acid sequences of VHs or VLs deduced from the gene sequencing, the correct sequence can be further judged and confirmed by LC-MS/MS analysis of the ascite mAb’s amino acid sequence. 

In this work, the predicted protein sequences of VHs and VLs were provided as the “antibody database”, and LC-MS/MS analysis was applied to the enzyme-digested peptides of the purified ascite mAb followed by the peptide coverage mapping based on the database. The mAb achieved 100% sequence coverage to the predicted VH sequence “SP-thiacloprid-C4C4 VH-99.88%” and the VL sequence “SP-thiacloprid-C4C4 V-λ 100%”, which were defined as hapten-specific sequences. The predicted VH sequence “SP-thiacloprid-C4C4 VH-0.11%” was not completely overlapped, meaning it was a contaminative sequence. This highlights the limitation of direct de novo mAb sequencing only based on LC-MS/MS due to the lack of comprehensive gene information, which causes difficulty related to the spectra interpretation and obscure in de novo assemblage of protein sequences. Sosthene et al. and Babrak et al. reported analogous strategies to characterize the mAb VRs through PCR amplification and MS/MS in 2010 and 2017 [12,44], but the NGS complemented with specific PCR in this work has advantages over single-PCR amplification, as discussed above. Our proposed integrated approach based on LC-MS/MS combined with NGS is efficient, reliable, and general.

The rAbs expressed in vitro are always adopted for activity verification of the identified VH and VL sequences. However, in the previous validation tests, we found that the hapten-specific rAb fragments such as scFv and Fab expressed in *E. coli* or yeast cells showed lower activity or were not expressed at all. A recent study reported that Fab secreted from mammalian cell line was the most homogeneous as the parental Fab compared to *E. coli* and *Pichia pastoris* [29]. Moreover, full-length IgGs have the advantages of dimeric epitope binding sites, which exhibited higher avidity than the antibody fragments. For instance, the full-length IgG expressed by the mammalian cell line has shown a rate of relative binding affinity to the antigen that is about 10 times higher than parental Fab [30]. Therefore, in this study we used the full-length IgG produced in the mammalian cell expression system for functional verification, which in return demonstrated the reliability of the proposed approach for VR sequence characterization. Excitingly, a novel anti-thiacloprid full-length rAb was achieved, with good performance close to that of the parental mAb, as evaluated by ELISA and GICS.

Furthermore, in silico docking simulation also demonstrated that the identified VH and VL were accurate and the VH–VL pairing was correct. Additionally, we successfully discovered that Ser52 (H-CDR2), Trp98 (L-CDR3), and Trp93 (L-CDR3) residues predominantly contributed to thiacloprid-specific recognition, mainly through hydrogen bonds and the CH–π interaction. Singly altering 3 key amino acids with alanine prevented or decreased the binding affinity of the mutated rAb. Computational approaches could help make progress in the in silico design of antibodies, for example through the physicochemical property improvement of antibodies, antibody structure modeling, and antibody–antigen complex prediction [45]. Understanding the structure-based determinants for the binding specificity and affinity will provide a theoretical basis for the correct interpretation of the antigen–antibody recognition mechanism and will also provide correct guidance for successful improvement of the antibody affinity [46]. The discovered key recognition sites also provide a theoretical basis for the establishment of effective immunoassays, i.e., the key amino acid sites that bind to the analyte should not be occupied or influenced.

## 4. Materials and Methods

### 4.1. SPR Evaluation of the mAb against Thiacloprid

Thiacloprid-C4C4 mAb was prepared according to the protocol described in our previous work [47]. The thiacloprid–bull serum albumin (BSA) conjugate reported by Zhenjiang Liu et al. [32] was used as the artificial immunogen to induce the immune response. For the initial and boosted immunizations, three female *BALB/c* mice (six-week-old) were respectively given 5 intramuscular injections with 100 μL of immunogen solution. The mouse that displayed the antiserum against thiacloprid was selected to donate the spleen for cell fusion with the myeloma cell. Via the classical hybridoma technology, a stable mAb-producing cell line C4C4 was obtained and used to generate mouse ascites. The subtype of mAb was determined as the IgG1 isotype with the λ chain. 

The amine coupling kit was used to immobilize anti-thiacloprid mAb onto the series S carboxymethyl dextran surface matrix 7 (CM7) sensor chip surface using a Biacore T200 instrument (GE Healthcare, Uppsala, Sweden). The selectivity evaluation and the direct binding kinetics and affinity of the mAb to 8 neonicotinoids and 2 negative controls (triazophos and chlorpyrifos) were evaluated by SPR technique (please refer to the Supplementary Materials for details of the experiment). 

### 4.2. Seeking of Multiple Accurate Sequences and Abundances of VR genes

#### 4.2.1. 5′-RACE and NGS of Antibody Transcriptome of Hybridoma

Sample preparation: Frozen hybridoma cells were removed from the liquid nitrogen tank and immediately placed into a 37 °C water bath. The thawed cells were washed once with PBS buffer and centrifuged at 200× *g* for 5 min. The cell precipitation was collected and used for subsequent RNA extraction.

Library preparation: Before constructing the NGS libraries, the usability of the 5′ universal forward adaptor primer and 3′reverse primers corresponding to the constant regions of each Ig-heavy chain (IgG\IgA\IgM\IgD\IgE) and Ig-light chain (Igκ\Igλ) was validated by using peripheral blood samples from mice. Each pair of primers successfully amplified about 700 bp antibody VR fragments of corresponding subtypes (Figure S11). RNA extraction of the mAb-secreting hybridoma was performed according to the instruction of the RNeasy Plus Mini Kit. The NGS libraries were prepared using the ImmuHub BCR profiling system (ImmuQuad Biotech, Hangzhou, China). Briefly, following the 5′-rapid amplification of cDNA ends (5′-RACE) unbiased amplification protocol, the validated 5′ universal forward adaptor primer and 3′ reverse primers (separately corresponding to the constant regions of 7 subtypes of the Ig-heavy/Ig-light chain) were used to facilitate PCR amplification in a less biased manner. The PCR amplified products of 5 subtypes of VH and 2 subtypes of VL were purified with the Agenecourt AMPure XP beads. To prepare final libraries compatible with Illumina^®^ sequencing platform, a second round of PCR was applied and the Illumina^®^ sequencing indices were added. The second-round PCR products were purified again. The final purified libraries were assessed by Agilent 2100 Bioanalyzer System (Agilent, Santa Clara, CA, USA) to detect the target peak of 600–900 bp and determine the molar concentration.

Sequencing and quality control: NGS was performed on an Illumina HiSeq 10x^®^ system with PE150 mode (Illumina, San Diego, CA, USA). The base call accuracy, measured by the Phred quality score (Q score), is the most common metric used to assess the accuracy of a sequencing platform. If Phred assigns a Q score of 30 (Q30) to a base, this means that the base call accuracy is 99.9%. When the sequencing quality reaches Q30, virtually all of the reads will be perfect. The Q30 scores of our libraries were all >80%.

Bioinformatics processing: The sequencing data were analyzed at a deep level using the ImmuHub^®^ BCR profiling system (ImmuQuad Biotech, Hangzhou, China). The immunoglobulin Basic Local Alignment Search Tool (IgBLAST) algorithm of National Center for Biotechnology Information (NCBI) was applied to raw sequencing data for the classification of V, D, J, and C gene segments under the mapping with ImMunoGeneTics database (IMGT^®^). The resulting nucleotide and amino acid sequences of CDR3 of VH and VL were determined. Those unproductive and aberrant sequences that were out-of-frame or with a stop codon were abandoned from the identified repertoires. The amount of each VH or VL clonotype was further defined by counting the numbers of clones with the same nucleotide sequence of CDR3. UMBs were used to correct errors that were generated from PCR amplifications and sequencing and to identify the original transcript molecule for each read. After parsing the UMB sequences from every read, the number of unique UMBs was summarized for each clone to get the counts for mRNA transcripts.

#### 4.2.2. Amplification of Accurate Full-Length Sequences of Related VRs by Sanger Sequencing 

Since NGS (HiSeq 10x^®^ system with PE150 mode) is unable to obtain the exact full-length sequence of a VH (360 bp) or VL (330 bp), specific primers (Table S2) were further designed according to the interested clones with IgG1 and λ subtypes from the NGS results. The full-length sequences of the VH and VL were amplified by specific PCR. Comparative experiments were performed at the same time (refer to Supplementary Materials and Table S3). The gene sequencing results were analyzed using IMGT (http://www.imgt.org/IMGT_vquest/input) and CDR classifications of antibodies were conducted by integrated database of antibody sequence and structure data (abYsis) (http://www.abysis.org) with the Kabat rule. Translated amino acid sequences were aligned separately by Protein BLAST in NCBI database (https://blast.ncbi.nlm.nih.gov/Blast.cgi). The amino acid sequences were aligned in each group using BioEdit software.

### 4.3. High-Resolution LC-MS/MS and Peptide Coverage Analysis

Here, 20 μg of the purified ascite mAb was firstly reduced with 10 mM dithiothreitol and alkylated with 20 mM iodoacetamide, then digested overnight by 5 endoproteinases (trypsin, pepsin, chymotrypsin, Asp-N, and Glu-C) separately in the right buffer with the appropriate pH according to the manufacturer’s protocol. The 3–4 μg peptides were analyzed by LC-MS/MS on a quadrupole orbitrap mass spectrometer (Q-Exactive, Thermo, Waltham, MA, USA) equipped with an ultra-high-performance LC separation system (Vanquish UHPLC, Thermo, Waltham, MA, USA) using higher energy collisional dissociation fragmentation. The LC-MS/MS parameters are supplied in the Supplementary Materials. The 5 raw digestion data files were used to perform sequence coverage analysis with PEAKS Studio X (Bioinformatics Solutions Inc., Waterloo, ON, Canada) based on the established “antibody VR sequence database”, including all mouse-derived VR sequences in the UniProt database and multiple interested sequences with abundance information from NGS. The identified amino acid sequences and nucleic acid sequences of VH and VL were deposited in GenBank with the accession number MT741909.

### 4.4. Performance Tests of the Expressed Full-Length rAb and the Parental mAb

#### 4.4.1. IC-ELISA

The hapten-specific recognition functions of the predicted VH and VL sequences were verified by the full-length rAb expressed in HEK 293(F) cells (supplied in the Supplementary Materials and Table S4). Here, 96-well ELISA plates were coated with 100 μL per well of thiacloprid-OVA at 0.03 mg/L for the parental mAb and 0.078 mg/L for the full-length rAb by overnight incubation at 4 °C. The following procedures were the same as described in our previous study [48], and details are given in the Supplementary Materials. 

#### 4.4.2. GICS Test

The GICS assembly and the preparation of immunoprobes (gold-labelled rabbit antimouse IgG antibodies) were prepared according to Lan et al. [49]. Briefly, thiacloprid-OVA was diluted to 3 mg/mL and fixed on a nitrocellulose membrane as the test line using a desktop film spraying machine (Hangan electronic technology, Hangzhou, China). Anti-thiacloprid mAb and the full-length rAb were respectively diluted to 50 and 25 μg/mL as the working concentrations. The reaction system included 3 μL of immunoprobes, 25 μL of antibody solution, and 25 μL of gradient-diluted thiacloprid solution (1.56–200 μg/L) or blank control (0.01 M, pH 7.4, PBS). The lowest concentration of pesticides at which the test line became almost invisible was defined as the LOD of the GICS test, as judged by the naked eye.

### 4.5. Discovery of the Key Amino Acids in the CDRs by Silico Analysis and Site-Directed Mutagenesis

The 3D structure of Fv was constructed by homology modeling (supplied in Supplementary Materials) and prepared by using the Structure Preparation module. The 2D structure of the thiacloprid was drawn in ChemBioDraw 2014 and converted to 3D conformation through energy minimization. A succession of three methods was used in Molecular Operating Environment v2018.01 (MOE; Chemical Computing Group Inc., Montreal, QC, Canada) to conduct an energy minimization procedure in the force field of AMBER10:EHT(Extended Hueckel Theory). When the gradient was extremely high, the method of steepest descent was used. When the gradient was sufficiently small (but still quite high), the method of conjugate gradient was used. Once the gradient was reasonable, the method of truncated Newton was used. The molecular docking simulation was conducted by using Dock module in MOE (refer to the Supplementary Materials for parameters setting) to predict binding free energy and to discover the key amino acids and main interactions that contributed to the stability of the small-molecule antibody complex. 

The revealed three key amino acids were mutated in a site-directed manner to alanine, and genes of three VH and VL mutants (VH-Ser52-A, VL-Trp93-A, VL-Trp98-A) were directly synthesized by the MasterGene^®^ platform (GENEWIZ, Suzhou, China). Three single-point mutated full-length rAbs were separately expressed in vitro by mammalian cells and their binding activities were analyzed by ELISAs. Here, 96-well plates were coated with 1 mg/L of thiacloprid-OVA (100 μL/well), followed by the reaction with individual rAbs diluted to final concentrations of 0.4–0.025 mg/L (100 μL/well). Each treatment was performed in triplicate, PBS buffer was set as the negative control, and the rAb_wild type was conducted as the positive control.

To further investigate the effect degree of the amino acid residue on the binding affinity of single-point-mutated rAb to thiacloprid, IC-ELISA was performed and IC_50_ was determined as described in Section 4.4.1.

## 5. Conclusions

In conclusion, we propose LC-MS/MS combined with NGS and specific PCR as an integrated and accurate approach for rapid profiling of hapten-specific VR gene sequences from hybridomas with diverse antibody genes. The expressed full-length rAb showed similar performance to the parental mAb against thiacloprid, indicating that the rAb could be further used to establish diverse immunoassays for rapid detection of thiacloprid residues in agricultural and environmental samples. The results of in silico molecular docking and single-site-directed alanine mutagenesis suggested that Trp93 (L-CDR3), Trp98 (L-CDR3), and Ser52 (H-CDR2) located in the antibody Fv were mainly responsible for the specific recognition of thiacloprid. This finding also proved that the approach of LC-MS/MS combined with NGS was reliably able to identify VH and VL sequences, as well as the core amino acids.

## Figures and Tables

**Table 1:** Comparison of the sensitivity of the anti-thiacloprid rAb with other reported polyclonal antibodies (pAbs) and mAbs.

Type of Antibody	Detection Method	IC_50_ (μg/L)	Year Reference
pAb	IC-TRFIA ^a^	1.90	2013, [31]
pAb	IC-ELISA	10.00	2013, [32]
pAb	CL-ELISA ^b^	30.90	2013, [33]
mAb	IC-ELISA	26.30	2015, [34]
mAb	IC-ELISA	2.31	2019, [35]
mAb	IC-ELISA	0.46	this study
full-length rAb	IC-ELISA	0.73	this study

^a^ Indirect competitive time-resolved fluoroimmunoassay (IC-TRFIA). ^b^ Chemiluminescence (CL) ELISA.

## Supplementary Materials

Supplementary Materials can be found at https://www.mdpi.com/1422-0067/21/18/6857/s1. Figures and tables: Figure S1. Sensorgram of anti-thiacloprid mAb immobilization. Figure S2. Low molecular weight (LMW) selectivity screening of the anti-thiacloprid mAb measured by SPR. Figure S3. Kinetics and affinity of anti-thiacloprid mAb with other neonicotinoid pesticides measured by SPR. Table S1. Detailed clone types and abundances of VH, Vλ, and Vκ in the hybridoma-C4C4 outputted by NGS. Figure S4. Total ion current (TIC) profiles of peptides produced by the digestion of mAb by five endoproteinases. Figure S5. (A) Identified peptides of anti-thiacloprid mAb by LC-MS/MS were assembled and 100% mapped to the predicted VH sequence “SP-thiacloprid-C4C4 VH-99.88%”. (B) Identified peptides of anti-thiacloprid mAb by LC-MS/MS were assembled and 100% mapped to the predicted VL sequence “SP-thiacloprid-C4C4 V-λ 100%”. Figure S6. (A) PCR amplification products of antibody VR fragments with homologous arms of expression plasmid. (B) SDS-PAGE of the rAb expressed in HEK 293 cells. Figure S7. Scheme of the recombinant plasmids for expression of the full-length IgG. Figure S8. Cross-reactivity of the parental mAb and the full-length rAb to thiacloprid and its structural analogues. Figure S9. Identity alignments of FRs with templates. Figure S10. (A) The 3D structure model of the variable region. (B) Ramachandran plot for model quality validation. (C) Verified 3D score plot for model quality validation. Figure S11. Validation of subtype-specific primers using peripheral blood samples of mice. Table S2. Specific primers designed based on NGS results used to amplify the exact sequences of VRs of interested clones. Table S3. Primer set 1 used to amplify VH and VL. Table S4. Primers used to amplify VH and VL with homologous arms to the expression vectors. Description of Results: Full-length IgG expressed in HEK 293(F) mammalian cells. The key amino acids in the CDRs for specific binding of thiacloprid. Description of Materials and Methods: Reagents and materials, SPR evaluation of the mAb against thiacloprid, selectivity evaluation, kinetics and affinity, seeking of multiple accurate sequences and abundances of VR genes, amplification of accurate full-length sequences of interested VRs by Sanger sequencing, high-resolution LC-MS/MS and peptide coverage analysis, liquid chromatography, mass spectrometry, MS/MS data analysis, expression of full-length rAb in HEK 293(F) cells, construction and identification of recombinant expression vectors, cell culture and transient expression, performance tests of the expressed full-length rAb and mAb, IC-ELISA, discovery the key amino acids in the CDRs by silico analysis and site-directed mutagenesis, homology modeling of antibody Fv, model evaluation, molecular docking.

## Abbreviations

mAb	monoclonal antibody	SPR	surface plasmon resonance
rAb	recombinant antibody	LMW	low molecular weight
Ig	immunoglobulin	RU	resonance unit
CR	cross reactivity	UMB	unique molecular barcode
NGS	next-generation sequencing	IC-ELISA	indirect competitive ELISA
CDR	complementarity-determining region	κ	kappa
LOD	limit of detection	λ	lambda
VR, VH, VL	variable region, variable region of a heavy chain, and variable region of a light chain	GICS	gold immunochromatographic strip
LC-MS/MS	liquid chromatography coupled with tandem mass spectrometry	Ka, Kd, KD	association rate, dissociation rate, dissociation equilibrium constant
Fv	variable fragment	VDW	van der Waals
BSA	bull serum albumin	OVA	ovalbumin
CM7	carboxymethyl dextran surface matrix 7	Q score	quality score
BCR	B-cell receptor		
