# Supplementary material for: Characterization of Variable Region Genes and Discovery of Key Recognition Sites in the Complementarity Determining Regions of the Anti-Thiacloprid Monoclonal Antibody
Source: Int J Mol Sci. 2020 Sep 18;21(18):6857. doi: 10.3390/ijms21186857 (PMC7555632; doi:10.3390/ijms21186857)
Supplement: Supplementary file 1 [file ijms-21-06857-s001.pdf]

Supplementary Materials

Characterization of Variable Region Genes and Discovery of Key Recognition Sites in the Complementarity Determining Regions of the Anti-Thiacloprid Monoclonal Antibody

Pengyan Liu [1], Yuanhao Guo [1], Shasha Jiao [1], Yunyun Chang [1], Ying Liu [1,2], Rubing Zou [1], Yihua Liu [1,3,*], Mengli Chen [1,4], Yirong Guo [1,*] and Guonian Zhu [1]

1 Institute of Pesticide and Environmental Toxicology, Ministry of Agriculture Key Laboratory of Molecular Biology of Crop Pathogens and Insects, Zhejiang University, Hangzhou 310058, China; lpyainimen@163.com (P.L.); 21916108@zju.edu.cn (Y.G.); 21616183@zju.edu.cn (S.J.); 21716193@zju.edu.cn (Y.C.); 21416116@zju.edu.cn (Y.L.); zourubing@zju.edu.cn (R.Z.); cmlmeng@126.com (M.C.); zhugn@zju.edu.cn (G.Z.)
2 Department of Food Science and Nutrition, Zhejiang Key Laboratory for Agro-Food Processing, Zhejiang University, Hangzhou 310058, China
3 Research Institute of Subtropical Forestry, Chinese Academy of Forestry, Hangzhou 311400, China
4 Zhejiang Provincial Key Laboratory of Biometrology and Inspection & Quarantine, College of life sciences, China Jiliang University, Hangzhou 310018, China
* Correspondence: liuyh@caf.ac.cn (Y.L.); yirongguo@zju.edu.cn (Y.G.); Tel.: +86-571-63122616 (Y.L.); +86-571-88982683 (Y.G.)

Table of contents

Figures and tables 4

Figure S1. Sensorgram of anti-thiacloprid mAb immobilization. 4

Figure S2. Low molecular weight (LMW) selectivity screening of the anti-thiacloprid mAb measured by SPR. 4

Figure S3. Kinetics and affinity of anti-thiacloprid mAb with other neonicotinoid pesticides measured by SPR. 6

Table S1. Detailed clone types and abundances of VH, Vλ and Vκ in the hybridoma- C4C4 outputted by NGS 6

Figure S4. Total ion current (TIC) profiles of peptides produced by the digestion of mAb by five endoproteinases. 7

Figure S5-A. Identified peptides of anti-thiacloprid mAb by LC-MS/MS were assembled and 100% mapped to the predicted VH sequence "SP-thiacloprid-C4C4 VH-99.88%" 8

Figure S5-B. Identified peptides of anti-thiacloprid mAb by LC-MS/MS were assembled and 100% mapped to the predicted VL sequence "SP-thiacloprid-C4C4 V-λ 100%". 9

Figure S6. (A): PCR amplification products of antibody VR fragments with homologous arms of expression plasmids. (B): SDS-PAGE of the rAb expressed in HEK 293 cells.. 10

Figure S7. Scheme of the recombinant plasmids for expression of the full-length IgG. 10

Figure S8. Cross reactivity of the parental mAb and the full-length rAb to thiacloprid and its structural analogues. 10

Figure S9. Identity alignments of FRs with templates 11

Figure S10. (A) The 3D-structure model of the variable region [a]; (B) Ramachandran plot for model quality validation; (C) Verified 3D score plot for model quality validation 11

Figure S11. Validation of subtype specific primers using peripheral blood samples of mice. 12

Table S2. Specific primers designed based on next-generation sequencing (NGS) results used to amplify the exact sequences of VRs of interested clones 12

Table S3. Primer set 1 used to amplify VH and VL 12

Table S4. Primers used to amplify VH and VL with homologous arms to the expression vectors 14

Results 15

Full-length IgG expressed in HEK 293(F) mammalian cells 15

The key amino acids in the CDRs for specific binding of thiacloprid 15

Materials and Methods 16

Reagents and materials 16

SPR evaluation of the mAb against thiacloprid 16

Selectivity evaluation 16

Kinetics and affinity 17

Seeking of multiple accurate sequences and abundances of VR genes 17

Amplification of accurate full-length sequences of interested VRs by Sanger sequencing 17

High resolution LC-MS/MS and peptide coverage analysis 17

Liquid chromatography 18

Mass spectrometry 18

MS/MS data analysis 18

Expression of full-length rAb in HEK 293(F) cells 19

Construction and identification of recombinant expression vectors 19

Cell culture and transient expression 19

Performance tests of the expressed full-length rAb and mAb 20

IC-ELISA 20

Discovery the key amino acids in the CDRs by silico analysis and site directed mutagenesis 20

Homology modelling of antibody Fv 20

Model evaluation 21

Molecular docking 21

Figures and tables

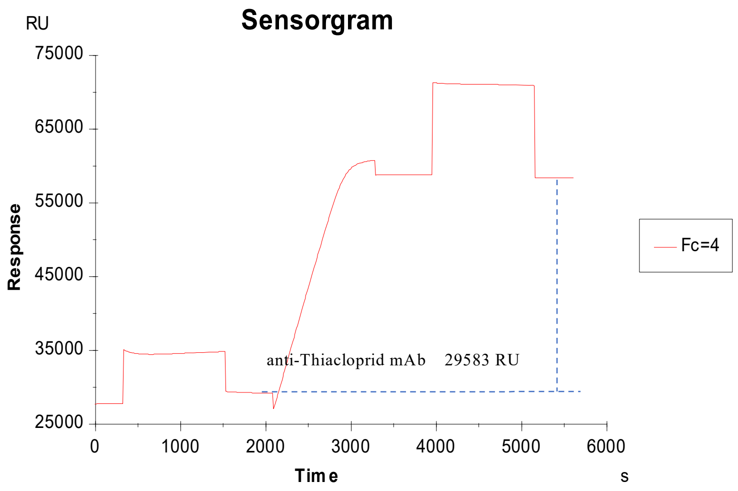

Figure S1. Sensorgram of anti-thiacloprid mAb immobilization.

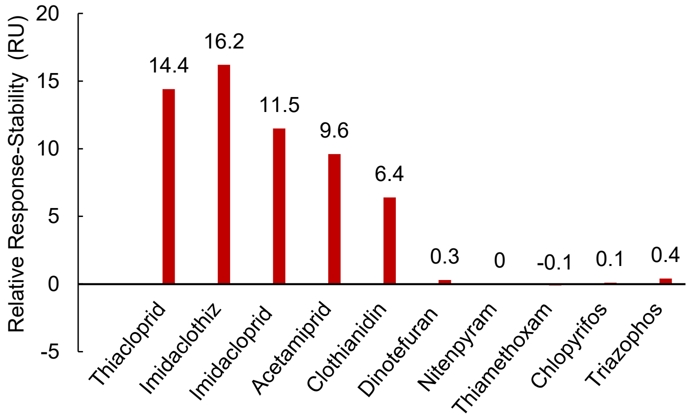

Figure S2. Low molecular weight (LMW) selectivity screening of the anti-thiacloprid mAb measured by SPR.

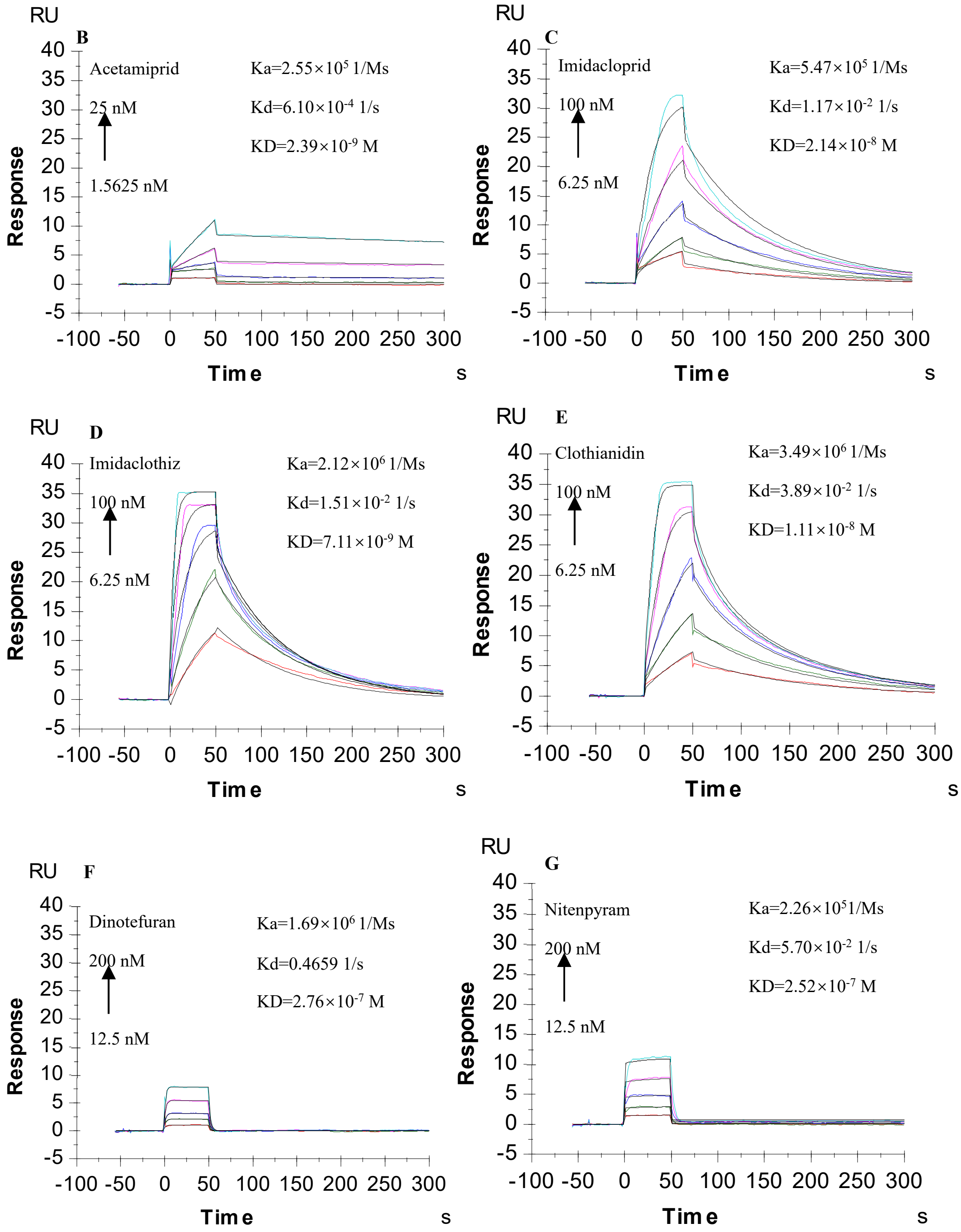
B
Acetamiprid
25 nM
1.5625 nM
Ka=2.55×10^5 1/Ms
Kd=6.10×10^-4 1/s
KD=2.39×10^-9 M
C
Imidacloprid
100 nM
6.25 nM
Ka=5.47×10^5 1/Ms
Kd=1.17×10^-2 1/s
KD=2.14×10^-8 M
D
Imidaclothiz
100 nM
6.25 nM
Ka=2.12×10^6 1/Ms
Kd=1.51×10^-2 1/s
KD=7.11×10^-9 M
E
Clothianidin
100 nM
6.25 nM
Ka=3.49×10^6 1/Ms
Kd=3.89×10^-2 1/s
KD=1.11×10^-8 M
F
Dinotefuran
200 nM
12.5 nM
Ka=1.69×10^6 1/Ms
Kd=0.4659 1/s
KD=2.76×10^-7 M
G
Nitenpyram
200 nM
12.5 nM
Ka=2.26×10^5 1/Ms
Kd=5.70×10^-2 1/s
KD=2.52×10^-7 M
RU
Response
Time
s

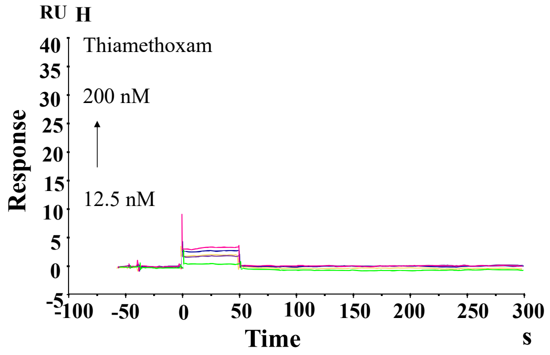

Figure S3. Kinetics and affinity of anti-thiacloprid mAb with other neonicotinoid pesticides measured by SPR.

Resonance units (RU) were double subtracted by the reference-flow cell signal and the buffer-control signal.

Table S1. Detailed clone types and abundances of VH, Vλ and Vκ in the hybridoma- C4C4 outputted by NGS

	Clone Count	Clone Abundance	CDR3 Gene Sequence	CDR3 Amino Acid Sequence
Thiacloprid-C4C4-λ	11712	1	TGTGCTCTGTGGTTCGGCAACCTTTGGGTGTTC	CALWFGNLWVF
Thiacloprid-C4C4-κ	247	0.980158	TGCGTGCAAGGTTCACATTTTCCTCACACGTTC	CVQGSHFPHTF
	1	0.003968	TGCCAGCAGTTTACTACTTCCCCATCTACGTTC	CQQFTTSPSTF
	1	0.003968	TGTCAGCACATTAGGGAGACGTTC	CQHIRETF
	1	0.003968	TGTCAGCACAGAGCTTACACGTTC	CQHRAYTF
	1	0.003968	TGTCAGGGAGCTTACACGTTC	CQGAYTF
	1	0.003968	TGTCAGCACACGTTC	CQHTF
Thiacloprid-C4C4-H	45188	0.998784	TGTGCTCGGATAACTTACCCCTTCTTTCCTATGGATTACTGG	CARITYPFFPMDYW
	50	0.001105	TGTTCAAGAGGGGGGCTTTACTATGATTACGACGCCTGGCTTGGTTACTGG	CSRGGLYYDYDAWLGYW
	1	2.21E-05	TGTGCTCGGATAACTTCCTTCTTTCCTATGGATTACTGG	CARITSFFPMDYW

	1	2.21E-05	TGTGCTCGGATAACTTACCCCTTTCCTATGGATTACTGG	CARITYPFPMDYW
	1	2.21E-05	TGTGCTCGGATAACTTTCTTTCCTATGGATTACTGG	CARITFFPMDYW
	1	2.21E-05	TGTGCTCGGATCTTTCCTATGGATTACTGG	CARIFPMDYW
	1	2.21E-05	TGTGCTCGGATAACTTACCCCTTCTTTTGG	CARITYPFFW

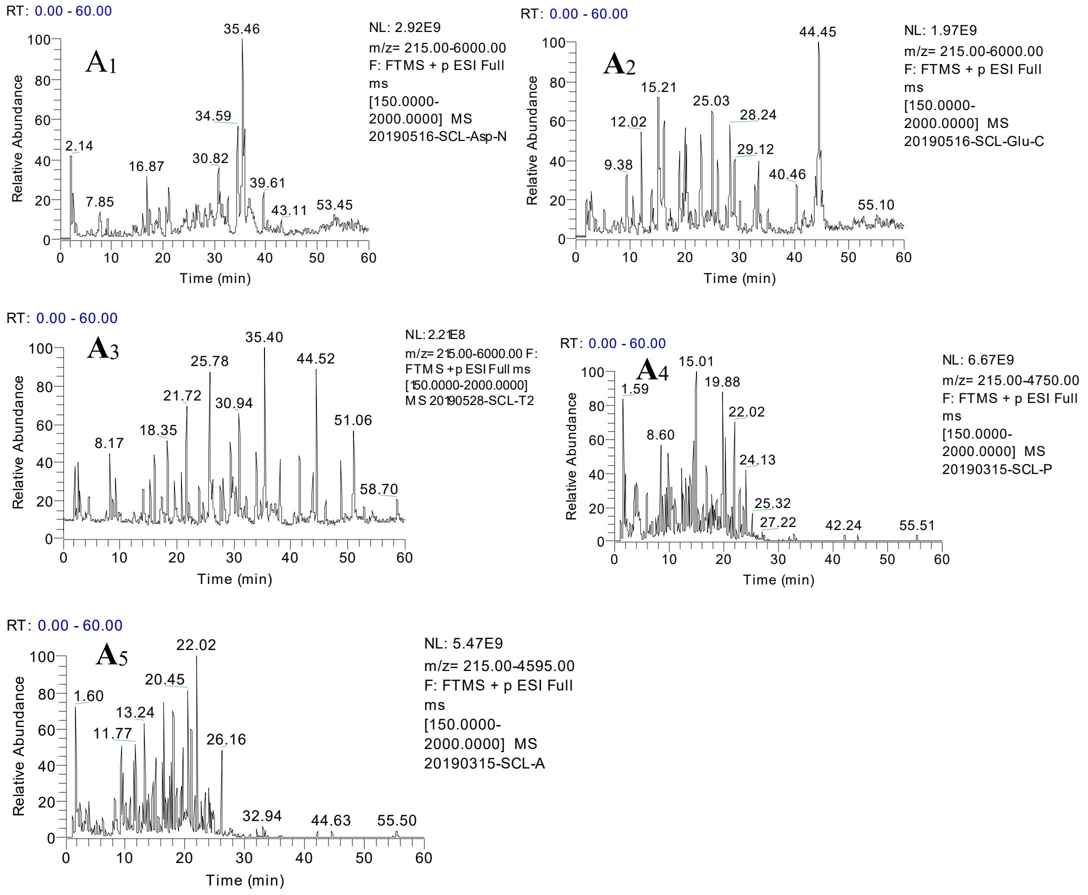

Figure S4. Total ion current (TIC) profiles of peptides produced by the digestion of mAb by five endoproteinases. A1-A5: mAb digested by ASP-N, Glu-C, trypsin, pepsin and chymotrypsin, respectively.

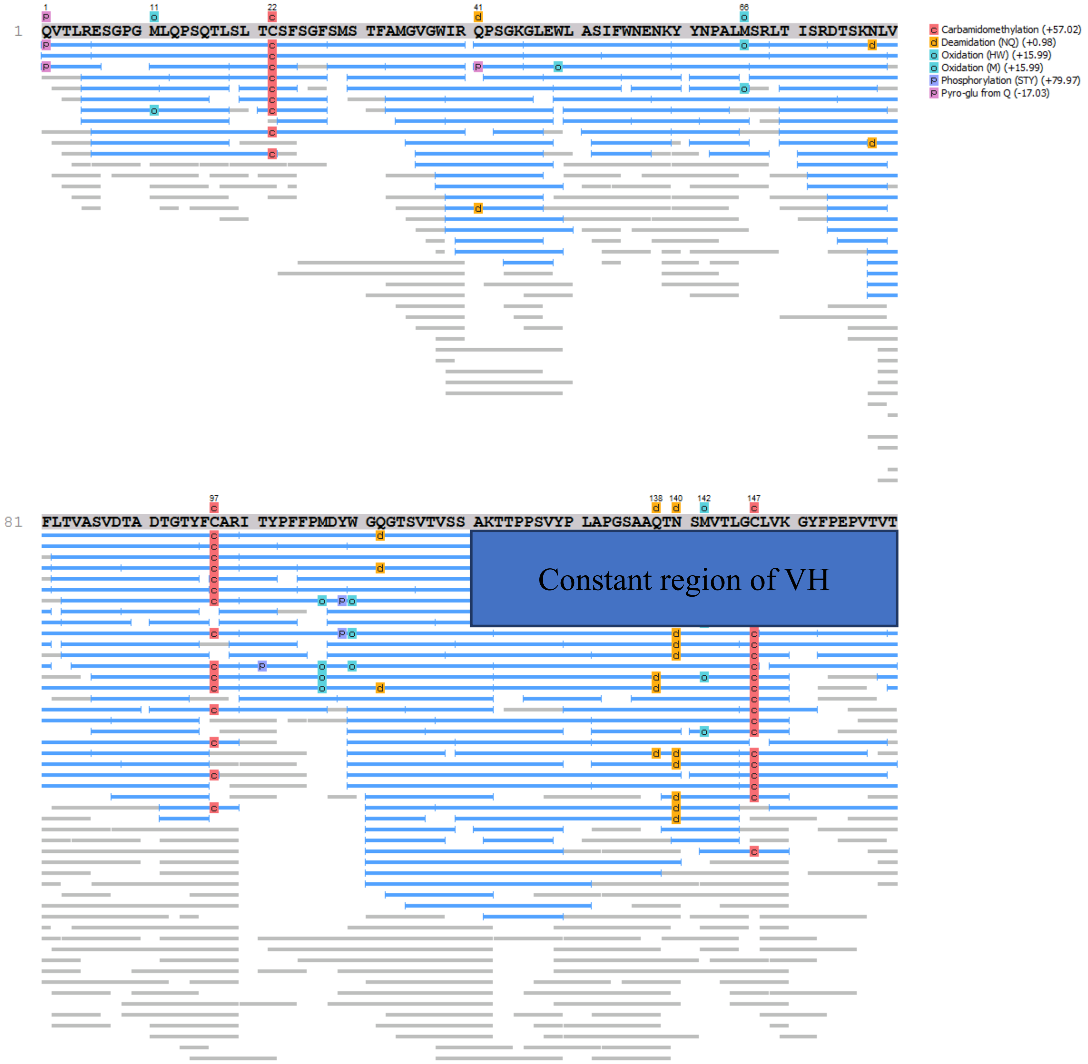

Figure S5-A. Identified peptides of anti-thiacloprid mAb by LC-MS/MS were assembled and 100% mapped to the predicted VH sequence "SP-thiacloprid-C4C4 VH-99.88%".

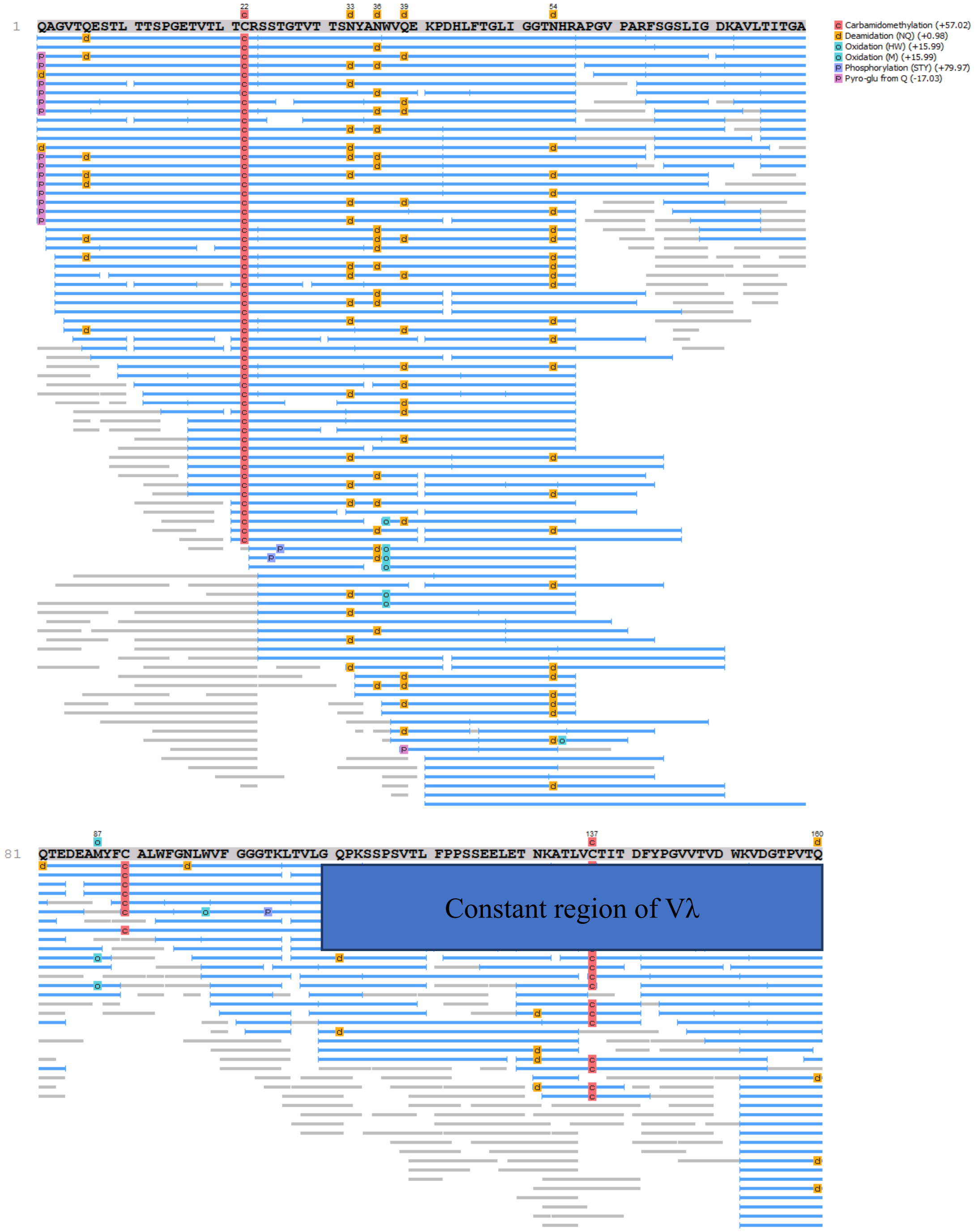

Figure S5-B. Identified peptides of anti-thiacloprid mAb by LC-MS/MS were assembled and 100% mapped to the predicted VL sequence "SP-thiacloprid-C4C4 V-λ 100%".

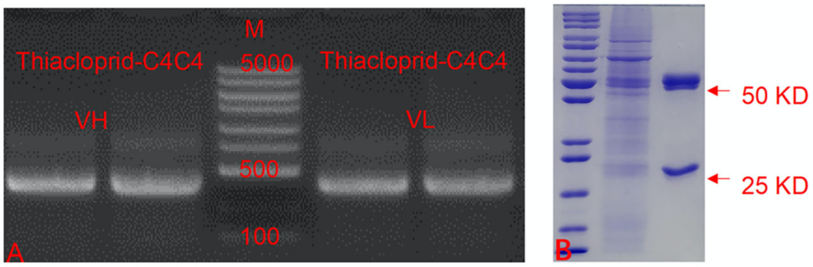

Figure S6. (A): PCR amplification products of antibody VR fragments with homologous arms of expression plasmids. M: 5000 DNA Marker map. (B): SDS-PAGE of the rAb expressed in HEK 293 cells. Left: 200 KD Marker, middle: supernatant of serum-free medium, right: heavy and light chains of purified full-length rAb.

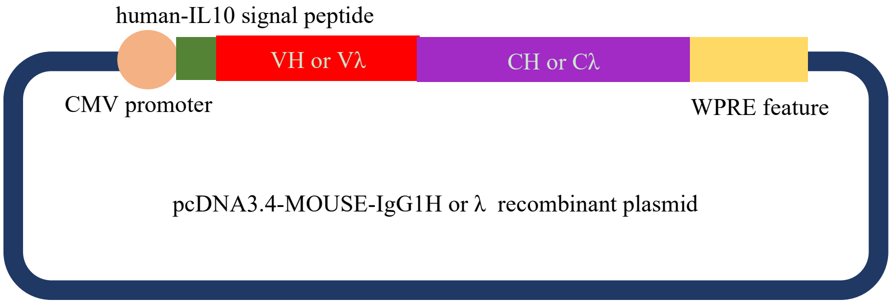

Figure S7. Scheme of the recombinant plasmids for expression of the full-length IgG.

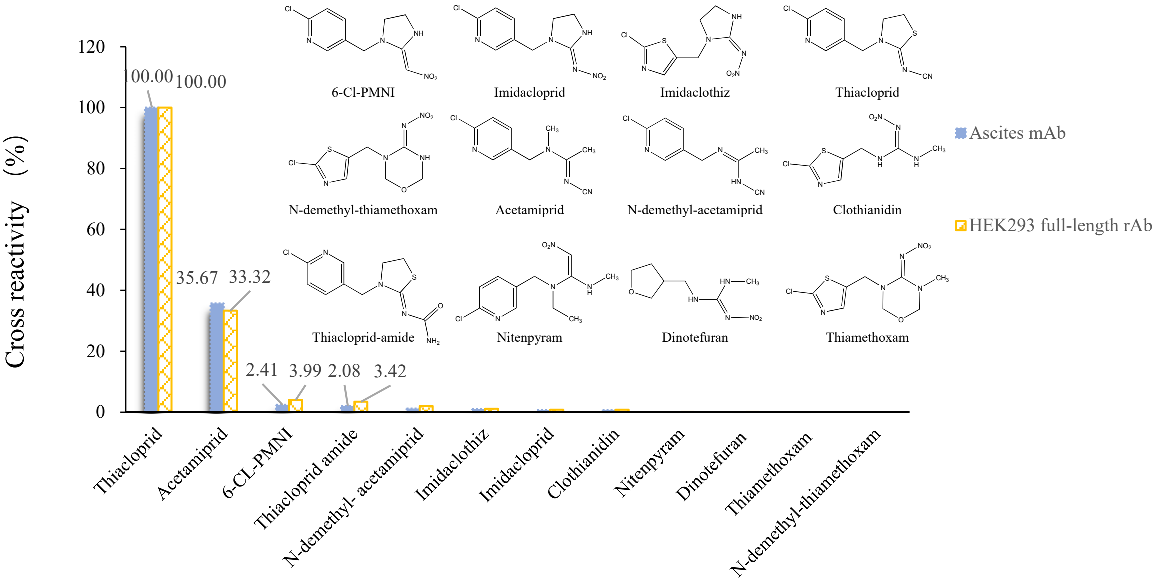

Figure S8. Cross reactivity of the parental mAb and the full-length rAb to thiacloprid and its structural analogues.

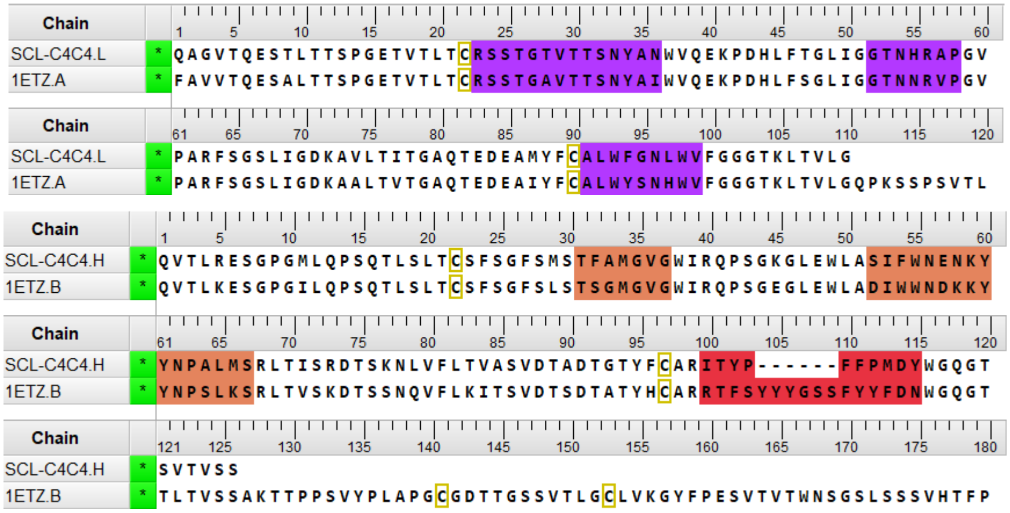

Figure S9. Identity alignments of FRs with templates.

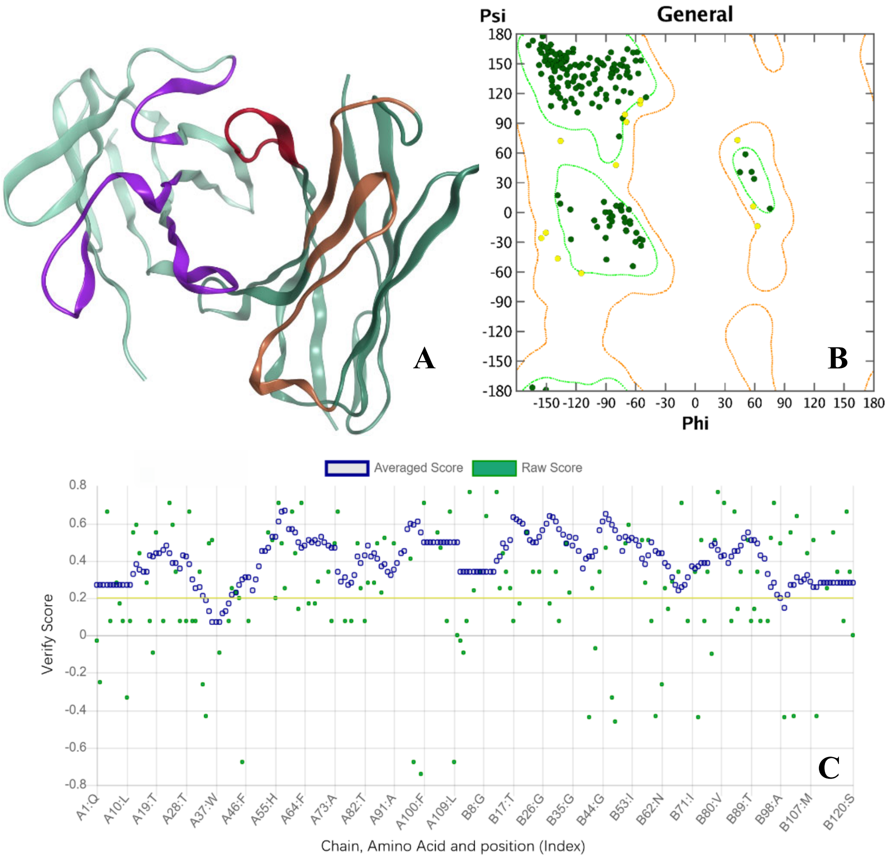

Figure S10. (A) The 3D-structure model of the variable region [a]; (B) Ramachandran plot for model quality validation; (C) Verified 3D score plot for model quality validation.

[a]: Variable region was shown in ribbons and colored in dark green (Framework, FR), purple (VL-CDR1/2/3), brown (VH-CDR1/2) and red (VH-CDR3).

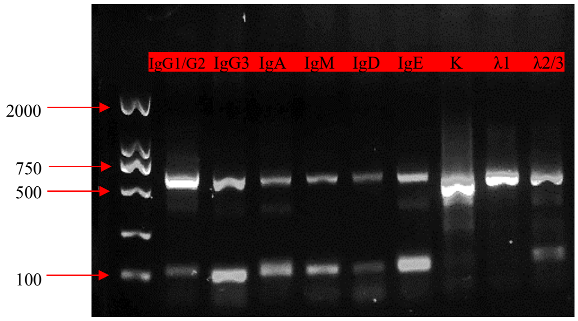

Figure S11. Validation of subtype specific primers using peripheral blood samples of mice. Forward primers were universal adaptor primer, reverse primers were designed at the constant regions of each subtype of the antibody Ig-Heavy chain (IgG\IgA\IgM\IgD\IgE) and Ig-Light chain (Igκ\Igλ) separately.

Table S2. Specific primers designed based on next-generation sequencing (NGS) results used to amplify the exact sequences of VRs of interested clones

	Forward primer (F) 5'-3'	Reverse primer (R) 5'-3'
SP-thiacloprid-C4C4 VH-0.11%	CAGGTCAAGCTGCAGCAGTC	TGAGGAGACGGTGACCGTGG
SP-thiacloprid-C4C4 VH-99.88%	CAGGTTACTCTGAGAGAGTC	TGAGGAGACGGTGACTGAGG
SP-thiacloprid-C4C4 V-λ 100%	CAGGCTGGTGTGACTCAGGA	TAGGACAGTCAGTTTGGTTC

Table S3. Primer set 1 used to amplify VH and VL [a]

	Primer name	Amino acid position	Sequence (5'-3')
VH-F	HB1	1-7	GAK GTR MAG CTT CAC GAG TC
	HB2	1-7	GAG GTB CAG CTB CAG CAG TC
	HB3	1-7	CAG GTG CAG CTG AAG SAR TC
	HB4	1-7	GAG GTC CAR CTG CAA CAR TC

	HB5	1-7	CAG GTY CAG CTB CAG CAR TC
	HB6	1-7	CAG GTY CAR CTG CAG CAR TC
	HB7	1-7	CAG GTC CAG GTG AAG CAR TC
	HB8	1-7	GAG GTG AAS STG GTG GAR TC
	HB9	1-7	GAV GTG AWG STG GTG GAG TC
	HB10	1-7	GAG GTG CAG STG GTG GAR TC
	HB11	1-7	GAK GTG CAM CTG GTG GAR TC
	HB12	1-7	GAG GTG AAG CTG ATG GAR TC
	HB13	1-7	GAG GTG CAR CTT GTT GAR TC
	HB14	1-7	GAR GTR AAG CTT CTC CAR TC
	HB15	1-7	GAA GTG AAR STT GAG GAR TC
	HB16	1-7	CAG GTT ACT CTR AAA SAR TC
	HB17	1-7	CAG GTC CAA CTV CAG CAR CC
	HB18	1-7	GAT GTG AAC TTG GAA SAR TC
	HB19	1-7	GAG GTG AAG GTC ATC GAR TC
	VHF-MH1	1-7	SAR GTN MAG CTG SAG SAG TC
	VH1-5'	1-8	CAG GTS MAR CTG CAG SAG TCW GG
	VH-A-5'	1-8	GAG GTG AAG CTT CTC GAG TCT GG
	VH-B-5'	1-8	SAG GTS CAG CTG MAG GAG TCW GG
	VH-C-5'	1-8	GAG GTC CAG CTG CAA CAA TCT GG
	VH-D-5'	1-8	SAG GTY CAR CTK CAG CAG YCT GG
	VH-E-5'	1-8	GAR GTG AAG CTT GWG GAG TCT GG
	VH-F-5'	1-8	SAG GTG MAG CTK CAS SAR TCW GG
	MVHF1	1-8	GAK GTR CAG CTT CAG GAG TCR GGA
	MVHF2	1-8	CAG GTG CAG CTG AAG SAG TCW GGM
	MVHF3	1-8	SAG GTY CAG CTG CAR CAG TCW GGD
	MVHF4	1-8	SAG GTC CAR CTG CAG SAR YCT GGR
	MVHF5	1-8	GAG GTT CAG CTG CAG CAG TCT GGG
	MVHF6	1-8	GAR GTG AAG CTG GTG GAR TCT GGR
	MVHF7	1-8	GAG GTG AAG CTT CTC GAG TCT GGA
	MVHF8	1-8	GAR GTG AAG CTK GAK GAG WCT GR
	MVHF9	1-8	GAV GTG MWG CTK GTG GAG TCT GGK
	MVHF10	1-8	SAG GTY CAG CTK CAG CAG TCT GGA
37 TOTALS			
VH-R	HF1	113-107	CGA GGA AAC GGT GAC CGT GGT
	HF2	113-107	CGA GGA GAC TGT GAG AAT GGT
	HF3	113-107	CGC AGA GAC AGT GAC CAG AGT
	HF4	113-107	CGA GGA GAC GGT GAC TGA GGT

	VHR-MHJ	113-109	TGM RGA GAC RGT GA
	VH1-2-3'	113-103	TGA GGA GAC GGT GAC CGT GGT CCC TTG GCC
	VH-A-3'	113-106	TGA GGA GAC GGT GAC CAT GGT CCC
	VH-B-3'	113-106	TGA GGA GAC TGT GAG AGT GGT GCC
	VH-C-3'	113-106	TGC AGA GAC AGT GAC CAG ACT CCC
	VH-D-3'	113-106	TGA GGA GAC GGT GAC TGA GGT CCC
	VH-E-3'	113-109	TGM RGA GAC RGT GA
	MVHB1	121-114	GAC AGR TGG GGS TGT YGT TTT GGC
	MVHB2	121-114	GAC AGA TGG GGC TGT TGT TKT
	MVHB3	121-114	GAC ATT TGG GAA GGA CTG ACT CTC
13 TOTALS			
V λ-F	LBλ	1-8	GAT GCT CTT GTG ACT CAG GAA TC
	MVLF6λ- 1	1-8	CAR SYT GTK STS ACT CAG GAA TCT
	MVLF6λ- 2	1-8	CAR SYT GTK STS ACT CAG GCA TCT
	MVLF6λ- 4	1-8	CAR SYT GTK STS ACT CAG TCA TCT
4 TOTALS			
V λ-R	LF λ	108-102	ACC TAG GAC AGT CAG TTT GG
	MVLB4	107-102	CTG RCC TAG GAC AGT SAS YTT GGT
2 TOTALS			
R=A/G, Y=C/T, M=A/C, K=G/T, S=C/G, W= A/T, H= A/C/T, B= C/G/T, V=A/C/G, D=A/G/T,			

[a] Forward primer (VH-F, VL-F) were designed at the beginning of the FR1 of variable region and reverse primers (VH-R, VL-R) at the end of the FR4 of variable region.

Table S4. Primers used to amplify VH and VL with homologous arms to the expression vectors

	F (5'-3')		R (5'-3')
Heavy chain-Signal peptide	CTCCTGACTGGGGTGAGGGCC + variable region sequence	IgG1-CH	AGATGGGGGTGTCGTTTTAGC + variable region sequence
λ chain-Signal peptide	CTCCTGACTGGGGTGAGGGCC +variable region sequence	λ-CL	TGGCGAAGACTTGGGCTGGCC + variable region sequence

Results

Full-length IgG expressed in HEK 293(F) mammalian cells

The VR gene fragments of antibody containing homologous arm of expression vectors were amplified from cloning vector carrying correct sequences of VRs, the gel electrophoresis results showed the apparent band of PCR products were near 400 bp (Fig. S6A), which were consistent with the theoretical calculation. Then the purified PCR products were linked to expression vectors pcDNA3.4-MOUSE-IgG1-CH and pcDNA3.4-MOUSE-Cλ to produce the recombinant plasmids separately, as shown in the sketch map Fig. S7. Followed by transient expression of the positive recombinant plasmid in mammalian cells HEK 293(F), 12% SDS-PAGE of the purified recombinant full-length antibody was displayed in Fig. S6B. The heavy chain band of 50 KD and the light chain band of 25 KD were clearly visible.

The key amino acids in the CDRs for specific binding of thiacloprid

The identity alignments of FRs with templates were showed in Fig. S9. For the FRs, the identities between queries and templates were 92.6% and 83.9% for VL and VH, respectively. For the six CDRs, the identities between queries and templates (L-CDR1: 1F4X.L, L-CDR2: 1ETZ.A, L-CDR3: 1ETZ.A, H-CDR1: 1ETZ.B, H-CDR2: 1ETZ.B, H-CDR3: 5GS0.F) are 100%, 71.4%, 66.7%, 75%, 62.5% and 50% for CDR-L1/L2/L3/H1/H2/H3, respectively. The homologous molded 3D-structure of anti-thiacloprid antibody VR displayed in Fig. S10-A. Ramachandran plot and verified 3D score plot were used to validate the quality of the constructed model. All residues located in the favored or allowed regions with no outlier observed (Fig. S10-B) and 96.09% of the residues had averaged 3D-1D score >= 0.2 (Fig. S10-C), which exhibited that the built model was of good quality and high reliability.

Materials and Methods

Reagents and materials

Standards of thiacloprid (# DRE-C17451000), imidaclothiz (# DRE-C14283850), acetamiprid (# DRE-C10013000), imidacloprid (# DRE-C14283700), dinotefuran (# DRE-C12820000), nitenpyram (# DRE-C15535000), thiamethoxam (# DRE-C17453000), clothianidin (# DRE-C11691700), N-demethyl-acetamiprid (# DRE-C10013200) were supplied by Dr. Ehrenstorfer. Thiacloprid amide (# P-1223S) and N-demethyl-thiamethoxam (# P-1266S) were purchased from AccuStandard. 6-Cl-PMNI (98.0%) was bought from Shanghai Shengnong Pesticide Co., Ltd. Rabbit anti-mouse IgG (whole molecule) antibody (# M7023), HRP-conjugated goat anti-mouse IgG (H+L) polyclonal antibody (# AP308P) and 5 endoproteinase (trypsin (# T8658), pepsin (# P7012), chymotrypsin (# C3142), Asp-N (# P3303) and Glu-C (# P6181)) were obtained from Sigma-Aldrich. RNeasy Plus Mini Kit was purchased from Qiagen (cat. # 74134). Gibson Assembly Master Mix (cat. # E2611S), restriction endonucleases HindIII (cat. # R0104S) and EcoRI (cat. # R0101S) were purchased from New England Biolabs. pcDNA3.4-MOUSE-IgG1-CH and pcDNA3.4-MOUSE-Cλ expression vector, HEK 293(F) mammalian cell line, serum-free medium and polyethyleneimine (PEI) were bought from Biointron (Jiangsu, China). Series S CM7 chip and amine coupling kit were purchased from GE Healthcare (Stockholm, Sweden); Agenecourt AMPure XP beads were supplied by Beckman Coulter, Inc (# A63882). All the other chemicals were analytical grade and ultrapure water was used throughout the experiments.

SPR evaluation of the mAb against thiacloprid

Selectivity evaluation

8 neonicotinoid pesticides and 2 negative controls (triazophos and chlorpyrifos) at 50 nM in HBS-EP+ running buffer were injected over the two flow cells at 25°C with a flow rate of 30 μL/min. The report point of the stability was set at the 10 s after the end of sample

injection. The 60 s' association for each sample followed by chip regeneration. Regeneration was conducted to sufficiently remove analyte by injecting 2 mM NaOH for 30 s at the flow rate of 30 μL/min.

Kinetics and affinity

The direct binding kinetics and affinity of thiacloprid and structural analogues were performed after 5 start-up injections with HBS-EP+. Two-fold dilution series of chemical compounds in HBS-EP+ and blank-control (zero concentration) were injected into the reference-flow cell and test-flow cell with the lowest concentration firstly. The different concentration solutions were injected at the flow rate of 30 μL/min for association with 60 s, followed by 600 s' dissociation. After each cycle of association phase and dissociation phase, the mAb-immobilized chip surface was regenerated with the injection of 2 mM NaOH for 30 s at the flow rate of 30 μL/min. Resonance units were double referenced by the reference-flow cell signal and the buffer-control signal using Biacore evaluation software 3.0 (GE Healthcare, USA). The double deducted curves were fitted to a simple 1:1 interaction model and the dissociation equilibrium constant (KD = Kd/Ka), dissociation rate (Kd) and association rate (Ka) were given in the final report.

Seeking of multiple accurate sequences and abundances of VR genes

Amplification of accurate full-length sequences of interested VRs by Sanger sequencing

Comparative experiment was performed using other primers designed with two strategies. Primer set 1: as shown in Table S2, the forward primers (VH-F, Vλ-F) were designed at the beginning of the framework region 1 (FR1) of VR and reverse primers (VH-R, Vλ-R) were designed at the end of the FR4 of VR (all degenerate oligonucleotides primers were mixed to use). Primer set 2 were commercially provided: the forward primers were designed at the beginning of the signal peptide before VR and reverse primers were designed at the beginning of the constant region.

High resolution LC-MS/MS and peptide coverage analysis

Liquid chromatography

Chromatographic column: Acquity UPLC BEH 300 C18 (Waters, American), mobile phase: 0.1% formic acid-aqueous solution (mobile phase A) and 0.1% formic acid-acetonitrile solution (mobile phase B), run time: 70 min, flow rate: 0.3 mL/min, column temperature: 35 °C, gradient elution mode: 0-5 min equilibrate the column with 5% B, 5-60 min peptide separation with 5-40% or 5-60% B, 61-65 min: flushing column with 100% B, 66-70 min: equilibrate the column with 5% B.

Mass spectrometry

Ion source: HESI+, Spray voltage: 3.8 KV, Sheath gas flow rate: 40, Aux gas flow rate: 10, Capillary temperature: 320 °C, S-lens RF level:55, Aux gas heater temperature: 350 °C, Data acquisition mode: full MS /dd-ms^2(top N), full MS mode: Resolution: 70000, AGC target: 1e6, maximum IT: 200 Ms, Scan range: 200 to 2000 m/z, spectrum data type: profile; dd MS^2 mode: Resolution: 17500, AGC target: 1e5, maximum IT: 100 Ms, Top N: 10, Isolation window: 1.6 m/z, (N)ce/stepped (N) CE: 28, spectrum data type: centroid; dd settings with Minimum AGC target: 8.00e3, peptide match: preferred, Dynamic exclusion: 10 S.

MS/MS data analysis

The successive steps were as follows: (1) data refinement: correct precursor data dependant analysis (DDA) with mass only, associate feature with chimera scan; (2) identification: error tolerance precursor mass: 10 ppm using monoisotopic mass, fragmentation: 0.05 Da, digest mode: semi-specific, maximum missed cleavages per peptide: 2; post-translational modification (PTM): carbamidomethylation of cysteines as a fixed modification, deamidation of glutamine and asparagine, oxidation of methionine and histidine and tryptophan, phosphorylation of serine and threonine and tyrosine, and pyro-glu from glutamine or glutamic acid as variable modifications, three modifications per peptide were allowed. Database: mouse Ig sequences from the UniPort

(https://www.uniprot.org) and supplemented with the predicted sequences of mAb determined by the NGS and sanger sequencing as described above, contaminant database: mouse proteome from the UniPort. 3) de novo sequencing: The Average Local Confidence (ALC) scores of peptide-spectrum matches (PSMs) identified must ≥ 50, database search: the scores of the PSMs identified with false discovery rate (FDR) 1%, PTM search with PTM score ≥13 as well as sequence variant and mutation search.

Expression of full-length rAb in HEK 293(F) cells

Construction and identification of recombinant expression vectors

The VR fragments with vector homologous arms were amplified from the positive cloning vectors, and the specific primers were showed in Table S3. Then, the VH and Vλ PCR fragments were subcloned into pcDNA3.4-MOUSE-IgG1-CH and pcDNA3.4-MOUSE-Cλ expression vector digested by Hind III and EcoR I to generate a recombinant plasmid, using Gibson cloning method. All recombinant clones were verified by DNA sequencing with primers (F: GATCGCCTGGAGACGCCATC; R: AGCGTAAAAGG AGCAACAT AGT).

Cell culture and transient expression

The HEK 293 (F) cells were cultured in a humidified incubator in serum-free medium at 37 °C with 120 rpm and 5% CO_2. After the initial cells have been subcloned for 2-3 generations, the portion of 1.5×10^6/mL cells were taken for instantaneous transfection in a sterile cone bottle based on the reported protocol with a minor modification. Total 90 μg of high-quality recombinant plasmids of antibody full-length heavy chain and light chain were mixed at a molar ratio of 2:3 and diluted in 6 mL of culture medium. 360 μL of 1 μg/μL PEI was diluted in 6 mL of culture medium in a polystyrol plate. Plasmid and PEI solution were mixed and incubated for 15-30 min at 37 °C, and the mixed transfection solution was added drop by drop to 180 mL medium solution with continuously shaking. After 7 days' culture, the supernatant was collected after centrifugation at 8000 rpm for 5 min. The

expressed rAb was purified by Protein A Resin Column and detected by SDS-PAGE.

Performance tests of the expressed full-length rAb and mAb

IC-ELISA

The coated plates were washed three times with PBST and did blocking with 2% milk-PBS at 37 °C for 1h. After the plate was washed two times with PBST, 50 μL per well of thiacloprid and structural analogues standards plus 50 μL per well of antibody with appropriate dilution in 2% milk-PBS were added into the micro-well plate. After 1h incubation at 37 °C, plates were washed three times again. Next, 100 μL per well of HRP-conjugated goat anti-mouse IgG (H+L) polyclonal antibody (diluted 1/40000 in 2% milk-PBS) was added, and plates were incubated 1h at 37 °C. The plates were washed four times, and the 3,3',5,5′-tetramethylbenzidine (TMB) was added to generate the signals of peroxidase activity. The enzymatic reaction was stopped with 2.5 M sulfuric acid after 15 min and the absorbance was immediately read at 450 nm. The relationship between the concentration of analyte and inhibition rate was analyzed using Origin 2017. The linear range to detect the analyte was defined as the analyte concentration toward 20%-80% inhibition. Cross reactivity (CR) %= [50% inhibitory concentration (analyte)]/[50% inhibitory concentration (structural analogues)]×100%.

Discovery the key amino acids in the CDRs by silico analysis and site directed mutagenesis

Homology modelling of antibody Fv

The software Molecular Operating Environment (MOE; Chemical Computing Group Inc., Montreal, Canada) v2018.01was used for constructing homology-based structure model of Fv. Amber10: EHT force field parameters, as implemented in MOE, was used. The 3D-structure of Fv was built using MOE Antibody Modeler by searching for appropriate templates of FRs and CDRs in the built-in antibody database (www.rcsb.org). The assigned templates for FR regions were 1ETZ.A for VL and 1ETZ.B for VH. The

assigned templates for CDR loops were L1: 1F4X.L, L2: 1ETZ.A, L3: 1ETZ.A, H1: 1ETZ.B, H2: 1ETZ.B, H3: 5GS0.F, respectively. The maximum number of main chain models was set to 5 and the number of side chain models per main chain was 25 by default. Up to 125 intermediate models were built for further scoring. GB/VI solvation was used for ranking the intermediate models. The model with the best GB/VI score was chosen for final energy minimization.

Model evaluation

The model quality was evaluated through the calculation of antibody Phi-Psi angles (aka. Ramachandran Plot), which was conducted by using the Protein Geometry module in MOE. The Verify-3D module of PROCHECK (https://servicesn.mbi.ucla.edu/PROCHECK/) was used to evaluate the stereochemical quality of each residue in the modeled antibody.

Molecular docking

The docking process adopts the flexible induced fit protocol for energy minimization, that the ligand was fully flexible during the conformation sampling process and side chains of the amino acids in combining pocket were also allowed to move. The protonation states and the orientations of the hydrogens were optimized by LigX, at the pH of 7 and temperature of 300 K. The docking process was performed under the force field of AMBER10: EHT along with an internal dielectric constant of 1 and external dielectric constant of 80, as well as an implicit solvation model of reaction field (R-field). The Triangle Matcher algorithm was used for the initial placement of 1000 returned conformations, and the top 100 conformations ranked by London dG scoring function were further refined through energy minimization followed by rescoring using Generalized Born/Volume Integral (GB/VI) solvation energy, a more accurate implicit solvent model scoring function, top-ranked poses were retained and the most representative pose was retrieved by visual inspection for further analysis. Finally, a representative complex

conformation was selected based on scoring and experience.
